# Cancer Incidence and Mortality Estimates in Latin America and the Caribbean: A Systematic Analysis of the GLOBOCAN 2022

**DOI:** 10.1158/2767-9764.CRC-25-0564

**Published:** 2025-12-29

**Authors:** Luís Felipe Leite, Lucas Diniz da Conceição, Erick F. Saldanha, Samuel Menezes, Andréia Cristina de Melo, Roberto Borea, Marcelo Corassa, Ana I. Velazquez, Andres Felipe Cardona, Oscar Arrieta, Eduardo Rios-Garcia, Luis Corrales, Christian Rolfo, Vladmir Cláudio Cordeiro de Lima

**Affiliations:** 1 Department of Medical Sciences, Universidade Federal Fluminense, Niterói, Brazil.; 2 Division of Medical Oncology and Hematology, Princess Margaret Cancer Centre, University Health Network, Toronto, Canada.; 3 Department of Medicine, University of Toronto, Toronto, Canada.; 4 Harvard T. H. Chan School of Public Health, Harvard University, Cambridge, Massachusetts.; 5 Department of Medical Sciences, Universidade Federal da Bahia, Salvador, Brazil.; 6 Division of Clinical Research and Technological Development, Brazilian National Cancer Institute, Rio de Janeiro, Brazil.; 7 Division of Medical Oncology, Department of Internal Medicine, The Arthur G. James Comprehensive Cancer Center, The Ohio State University Comprehensive Cancer Center, Columbus, Ohio.; 8 Thoracic Oncology Unit, BP – A Beneficência Portuguesa de São Paulo, São Paulo, Brazil.; 9 Division of Hematology/Oncology, University of California, San Francisco, San Francisco, California.; 10 Direction of Research, Science and Education, Luis Carlos Sarmiento Angulo Cancer Treatment and Research Center (CTIC), Bogotá, Colombia.; 11 Thoracic Oncology Unit, Instituto Nacional de Cancerología, Mexico City, Mexico.; 12 Medical Oncology, Centro de Investigación y Manejo del Cancer-CIMCA, San Jose, Costa Rica.; 13 Department of Medical Oncology, Thoracic Cancer Reference Center, A. C. Camargo Cancer Center, São Paulo, Brazil.

## Abstract

Cancer is a leading cause of death in Latin America and the Caribbean (LAC), and up-to-date estimates are essential to guide cancer policy. Using GLOBOCAN 2022 data, we analyzed cancer incidence and mortality across 32 LAC countries, calculated age-standardized rates, and assessed early-onset cancer (diagnosed at ages 15–50 years). Mortality-to-incidence ratios were used as a proxy for survival, joinpoint regression estimated annual percent change, and linear regression evaluated correlation between the Human Development Index and cancer indicators. In 2022, LAC recorded 1,551,060 new cancer cases (age-standardized incidence rate, 186.6 per 100,000) and 749,242 deaths (age-standardized mortality rate, 85.2 per 100,000). Prostate and breast cancers were the most common malignancies, whereas lung and breast cancers caused the highest mortality. Mortality declined for prostate cancer [annual percent change, −1.52; 95% confidence interval (CI), −1.80 to −1.24] and male lung cancer (−2.50; 95% CI, −2.68 to −2.33) but increased for female lung (+1.88; 95% CI, 1.71–2.05) and colorectal cancer (+2.48; 95% CI, 2.30–2.67). Human Development Index showed an inverse correlation with mortality-to-incidence ratio (*P* < 0.001), suggesting improved survival with higher development. Early-onset cancers represented 17% of new cases and 11% of deaths. These findings reveal a growing cancer burden and that persistent disparities in cancer epidemiology persist across LAC, highlighting the urgent need for targeted cancer control strategies and regional cancer control plans.

**Significance:**

These findings highlight the urgent need for equity-focused cancer control policies, improved early detection, and expanded access to essential cancer care in the region.

## Introduction

Cancer remains a critical challenge for global health systems, primarily driven by aging populations, increasing exposure to cancer risk factors, and enduring inequities in healthcare access (1). Worldwide, it is responsible for approximately 10 million deaths annually, with nearly 65% occurring in low- and middle-income countries (2). This geographic imbalance highlights a pressing public health paradox: the most significant cancer burden is carried by regions least equipped to manage it effectively. Despite substantial advancements in cancer prevention, early detection, and treatment modalities, disparities persist (3). In fact, cancer control strategies across Latin America and the Caribbean (LAC) were found to vary widely in their design and implementation, with persistent gaps in screening coverage, vaccination uptake, and equitable access to treatment services (4).

In LAC, the cancer landscape is complex and evolving rapidly, shaped by demographic shifts, urbanization, changing lifestyles, and enduring socioeconomic disparities (5). The region, home to more than 650 million people, about 8.4% of the global population, includes two of the 10 most populous nations, Brazil and Mexico. Since the 1960s, population growth has slowed steadily, with a growth rate of 0.9% in 2020, reflecting a demographic transition marked by declining fertility and increased longevity. Life expectancy at birth increased from 50 years in the early 1950s to 76 years in 2020 and is expected to continue climbing. The proportion of the population aged 65 years and older has more than doubled since 1980 and is projected to reach 20% by 2050 and 30% by the end of the century. These changes are especially pronounced among women, who significantly outnumber men in older age groups (128 women per 100 men aged 65 years and over and 157 per 100 among those 80 years of age and older; refs. 6–8). The anticipated demographic transitions heighten the urgency for comprehensive regional strategies to manage this growing burden effectively (9, 10).

This work comprehensively assesses the current cancer burden in LAC. It analyzes recent epidemiologic data, evaluates early-onset cancer trends, and projects future cancer incidence and mortality. Additionally, we aim to identify gaps in cancer care delivery and infrastructure and propose strategic recommendations for mitigating the anticipated increase in the cancer burden.

## Materials and Methods

### Study overview

This population-based analysis aimed to provide updated estimates of the cancer mortality and early-onset disease burden (defined as those diagnosed between the ages of 15 and 50 years) within LAC. Cancer incidence and mortality data were extracted from the publicly available GLOBOCAN 2022 database maintained by the International Agency for Research on Cancer (11, 12). The analysis comprised 32 countries from the LAC region, allowing for comprehensive regional assessments and cross-country comparisons. Data from the United States were included exclusively for contextual comparison given its comprehensive cancer registry coverage.

Population-specific estimates and demographic projections were obtained from the United Nations (UN) World Population Prospects 2019 forecasts to support age-specific analyses (13). These data were stratified into 5-year age groups within the defined range (15–49 years) to evaluate early-onset cancer patterns properly. Age standardization of incidence and mortality rates was conducted per 100,000 person-years using the Segi–Doll World Standard Population to enable comparability across different global populations and eliminate age-related confounding effects.

The included countries’ development levels were assessed using the Human Development Index (HDI), which integrates life expectancy, education, and gross national income indicators and allows categorization into four tiers (low, medium, high, and very high). This categorization facilitated the assessment of potential associations between cancer burden and country-level socioeconomic development. Additionally, all analyses were stratified by sex to account for sex-specific differences in cancer incidence and mortality.

### Statistical analysis

Estimates included new cancer cases, deaths, and corresponding age-standardized rates per 100,000 person-years, specifically the age-standardized rates of incidence and mortality in 2022 were calculated, stratified by sex and country for all cancers combined and by individual cancer as well (14).

Future trend analysis (1990–2022) was conducted for all ages and early-onset cancers (15–49 years) using integrated UN demographic forecasts with observed age-specific cancer incidence and mortality rates from 2022. Projections for the year 2050 were calculated under the assumption that the age-specific incidence and mortality rates observed in 2022 would remain unchanged. Therefore, projected changes in cases and deaths reflect demographic changes alone, including population growth and aging. To further evaluate cancer outcomes with regard to diagnosis rates, we calculated each country’s mortality-to-incidence ratio (MIR) as a proxy for cancer survival and health system performance.

Additionally, to provide insights into plausible future scenarios beyond demographic effects, we modeled seven hypothetical scenarios that assessed the potential impacts of annual increases or decreases (0%, ±1%, ±2%, and ±3%) in age-specific incidence and mortality rates from 2022 to 2050. These parameters were selected according to previously published modeling approaches (15). Data manipulation and visualization were done using the R packages “dplyr” and “ggplot2.” Finally, past temporal trends until 2022 were analyzed through jointpoint regression, exploring annual percent changes (APC).

### Ethical considerations

Ethical approval was not required for this study as it involved publicly available, deidentified secondary data without human subject’s research components. Nevertheless, the study adheres to the ethical guidelines of the Declaration of Helsinki.

## Results

### Incidence and mortality

**Figure 1.**
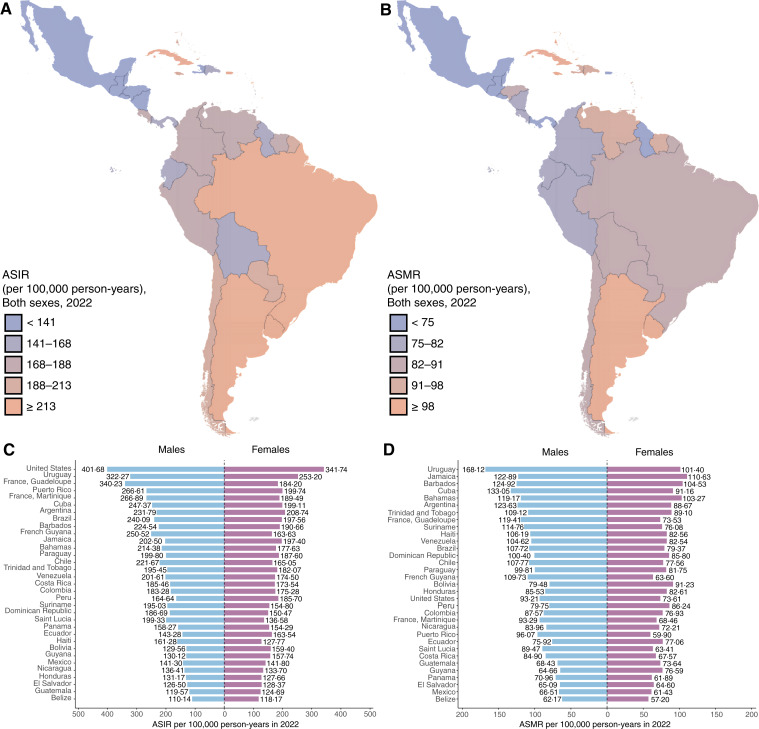
ASIR and ASMR in 2022 for all ages and countries. **A,** LAC map of ASIR for both sexes. **B,** LAC map of ASMR for both sexes. **C,** Bar plot of ASIR stratified by sex and country. **D,** Bar plot of ASMR stratified by sex and country.

**Figure 2.**
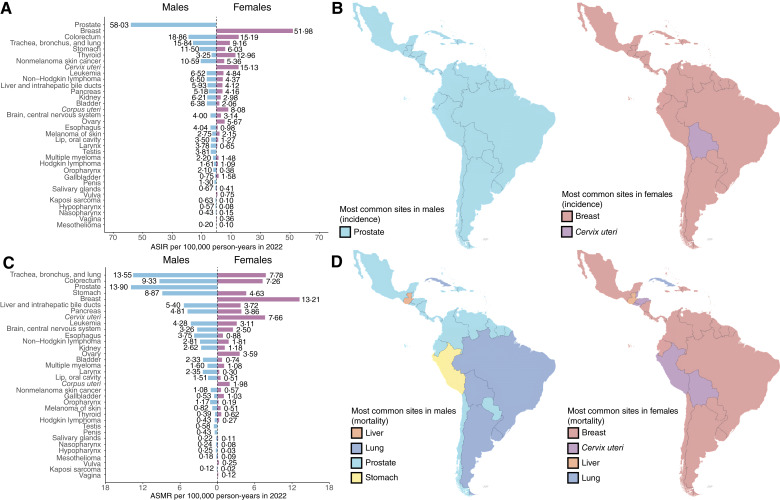
Cancer types by ASIR and ASMR in 2022 across all age groups. **A,** Bar plot of ASIR stratified by sex and cancer type. **B,** Sex-stratified maps of LAC showing the most common incidence sites by country. **C,** Bar plot of ASMR stratified by sex and cancer type. **D,** Sex-stratified maps of LAC showing the most common mortality sites by country.

In 2022, LAC recorded 1,551,060 new cancer cases. Brazil had the highest number (627,193), followed by Mexico (207,154) and Argentina (133,420; Table 1; Fig. 1). Among men, Brazil reported 319,711 new cases, whereas Argentina had the highest age-standardized incidence rate (ASIR; 322.3 per 100,000). Among women, Brazil also showed the largest absolute incidence (307,482), and Uruguay had the highest ASIR (253.2 per 100,000). The most frequent cancers were prostate (ASIR = 58 per 100,000) and colorectal (ASIR = 18.9 per 100,000) in men and breast (ASIR = 41.7 per 100,000), colorectal (ASIR = 15.2 per 100,000), and cervical (ASIR = 15.1 per 100,000) in women. Breast cancer was the leading malignancy among women in all countries except Bolivia, where cervical cancer predominated (Fig. 2; Table 2).

**Table 1.** Absolute counts of cancer cases and deaths, ASIR and ASMR rates, and HDI values, stratified by country and sex across all age groups for the year 2022.

Country	Male				Female				Total		HDI
	Absolute incidence	ASIR	Absolute mortality	ASMR	Absolute incidence	ASIR	Absolute mortality	ASMR	Absolute incidence	Absolute mortality	Value
Argentina	65,040	231.8	36,588	123.6	68,380	208.7	33,663	88.7	133,420	70,251	0.849
The Bahamas	477	214.4	266	119.2	478	177.6	286	103.3	955	552	0.82
Barbados	578	224.5	381	124.9	542	190.7	357	104.5	1,120	738	0.809
Bolivia	7,692	129.6	4,958	79.5	9,887	159.4	6,057	91.2	17,579	11,015	0.698
Brazil	319,711	240.1	146,702	107.7	307,482	197.6	132,133	79.4	627,193	278,835	0.76
Belize	188	110.1	106	62.2	221	118.2	105	57.2	409	211	0.7
Chile	32,800	221.7	16,897	107.8	27,076	165.1	14,543	77.6	59,876	31,440	0.86
Colombia	56,224	183.3	27,816	87.6	61,396	175.3	28,903	76.9	117,620	56,719	0.758
Costa Rica	6,808	185.5	3,246	84.9	6,517	173.5	2,826	67.6	13,325	6,072	0.806
Cuba	26,892	247.4	15,854	133.1	22,796	199.1	11,870	91.2	49,688	27,724	0.764
Dominican Republic	10,735	186.7	6,176	100.4	9,436	150.5	5,568	85.8	20,171	11,744	0.766
Ecuador	13,890	143.3	7,658	75.9	16,998	163.5	8,500	77.1	30,888	16,158	0.765
El Salvador	4,205	126.5	2,290	65.1	5,594	128.4	3,002	64.6	9,799	5,292	0.674
French Guyana	341	250.5	138	109.7	248	163.6	95	63.6	589	233	0.79
France, Guadeloupe	1,312	340.2	533	119.4	811	184.2	419	73.5	2,123	952	0.86
Guatemala	7,783	119.6	4,544	68.4	10,018	124.7	5,825	73.6	17,801	10,369	0.629
Guyana	537	130.1	271	64.7	688	157.7	341	76.6	1,225	612	0.742
Haiti	7,028	161.3	4,595	106.2	6,832	127.8	4,419	82.6	13,860	9,014	0.552
Honduras	5,051	131.2	3,364	85.5	5,764	127.7	3,647	82.6	10,815	7,011	0.624
Jamaica	3,713	202.5	2,423	122.9	3,787	197.4	2,209	110.6	7,500	4,632	0.706
France, Martinique	1,175	266.9	459	93.3	882	189.5	427	68.5	2,057	886	0.854
Mexico	95,954	141.3	46,415	66.5	111,200	141.8	49,795	61.4	207,154	96,210	0.781
Nicaragua	3,763	136.4	2,306	84.0	4,646	133.7	2,516	72.2	8,409	4,822	0.669
Panama	4,156	158.3	1,963	71.0	4,197	154.3	1,807	61.9	8,353	3,770	0.82
Paraguay	6,966	199.8	3,532	99.8	6,817	187.6	3,049	81.8	13,783	6,581	0.731
Peru	33,724	164.6	16,790	79.8	39,103	185.7	19,144	86.2	72,827	35,934	0.762
Puerto Rico	7,486	266.6	3,212	96.1	6,292	199.7	2,584	59.9	13,778	5,796	N/A
Saint Lucia	265	199.3	132	89.5	183	136.6	91	63.4	448	223	0.725
Suriname	571	195.0	340	114.8	548	154.8	286	76.1	1,119	626	0.69
Trinidad and Tobago	1,969	195.5	1,169	109.1	1,962	182.1	1,052	89.1	3,931	2,221	0.814
Uruguay	8,579	322.3	4,920	168.1	8,238	253.2	4,060	101.4	16,817	8,980	0.83
Venezuela	31,374	201.6	16,346	104.6	31,573	174.5	15,391	82.5	62,947	31,737	0.699

**Table 2.** Absolute counts of cases and deaths with corresponding ASIR and ASMR rates, stratified by cancer type and sex across all age groups, for the year 2022.

Cancer type	ICD	Male				Female				Total	
		Absolute incidence	ASIR	Absolute mortality	ASMR	Absolute incidence	ASIR	Absolute mortality	ASMR	Absolute incidence	Absolute mortality
Lip, oral cavity	C00-06	13,098	3.5	5,697	1.5	6,153	1.3	2,625	0.51	19,251	8,322
Salivary glands	C07-08	2,549	0.67	881	0.22	1,870	0.41	548	0.11	4,419	1,429
Oropharynx	C09-10	7,766	2.1	4,368	1.2	1,726	0.38	925	0.19	9,492	5,293
Nasopharynx	C11	1,577	0.43	899	0.24	636	0.15	358	0.08	2,213	1,257
Hypopharynx	C12-13	2,139	0.57	949	0.25	361	0.08	143	0.03	2,500	1,092
Esophagus	C15	15,454	4.0	14,430	3.8	4,861	0.98	4,417	0.88	20,315	18,847
Stomach	C16	45,056	11.5	35,027	8.9	29,201	6.0	22,776	4.6	74,257	57,803
Colorectum	C18-C21	72,752	18.9	36,956	9.3	72,017	15.2	36,490	7.3	144,769	73,446
Liver and intrahepatic bile ducts	C22	22,889	5.9	21,059	5.4	19,775	4.1	18,200	3.7	42,664	39,259
Gallbladder	C23	2,988	0.76	2,105	0.53	7,437	1.6	4,938	1.0	10,425	7,043
Pancreas	C25	20,180	5.2	18,827	4.8	20,764	4.2	19,413	3.9	40,944	38,240
Larynx	C32	14,270	3.8	9,006	2.4	2,980	0.65	1,424	0.29	17,250	10,430
Trachea, bronchus, and lung	C33-34	61,777	15.8	53,311	13.5	43,175	9.2	37,239	7.8	104,952	90,550
Melanoma of skin	C43	10,679	2.8	3,280	0.83	9,599	2.2	2,560	0.51	20,278	5,840
Nonmelanoma skin cancer	C44	43,182	10.6	4,589	1.1	29,643	5.4	3,484	0.57	72,825	8,073
Mesothelioma	C45	767	0.20	710	0.18	441	0.10	399	0.09	1,208	1,109
Kaposi sarcoma	C46	2,387	0.63	452	0.12	426	0.10	98	0.02	2,813	550
Breast	C50	—	—	—	—	219,684	52.0	59,701	13.2	219,684	59,701
Vulva	C51	—	—	—	—	3,647	0.75	1,359	0.25	3,647	1,359
Vagina	C52	—	—	—	—	1,615	0.36	572	0.12	1,615	572
*Cervix uteri*	C53	—	—	—	—	63,056	15.1	33,443	7.7	63,056	33,443
*Corpus uteri*	C54	—	—	—	—	34,612	8.1	9,250	2.0	34,612	9,250
Ovary	C56	—	—	—	—	24,031	5.7	15,881	3.6	24,031	15,881
Penis	C60	5,185	1.3	1,674	0.43	—	—	—	—	5,185	1,674
Prostate	C61	225,316	58.0	60,792	13.9	—	—	—	—	225,316	60,792
Testis	C62	13,644	3.8	2,103	0.58	—	—	—	—	13,644	2,103
Kidney	C64	23,032	6.2	10,066	2.6	12,851	3.0	5,594	1.2	35,883	15,660
Bladder	C67	25,521	6.4	9,804	2.3	10,188	2.1	4,100	0.74	35,709	13,904
Brain, central nervous system	C70-72	14,296	4.0	11,901	3.3	12,651	3.1	10,595	2.5	26,947	22,496
Thyroid	C73	11,946	3.3	1,518	0.39	51,533	13.0	3,081	0.62	63,479	4,599
Hodgkin lymphoma	C81	5,774	1.6	1,619	0.43	4,259	1.1	1,212	0.27	10,033	2,831
Non–Hodgkin lymphoma	C82-86+C88	24,038	6.5	10,730	2.8	19,005	4.4	8,481	1.8	43,043	19,211
Multiple myeloma	C90	8,395	2.2	6,215	1.6	6,758	1.5	5,154	1.1	15,153	11,369
Leukemia	C91-95	22,504	6.5	15,670	4.3	18,421	4.8	12,942	3.1	40,925	28,612

**Figure 3.**
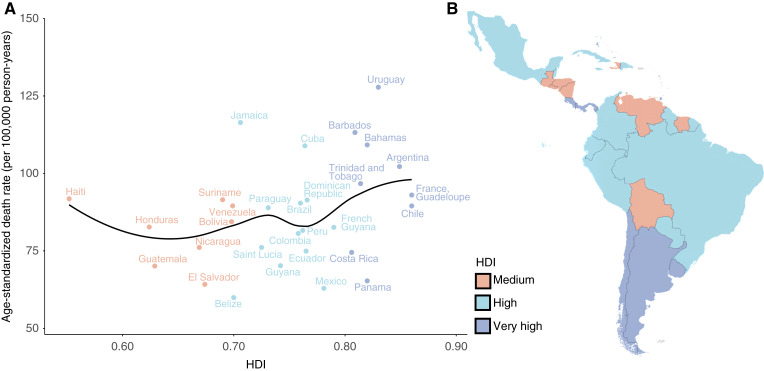
HDI and ASMR vs. HDI in 2022. **A,** Plot displaying the relationship between HDI and ASMR. **B,** Map of HDI distribution across LAC countries.

A total of 749,242 cancer deaths occurred in LAC in 2022. Brazil registered the highest number (278,835), followed by Mexico (96,210) and Argentina (70,251). Among men, Brazil had 146,702 deaths, but Uruguay reported the highest age-standardized mortality rate (ASMR; 168.1 per 100,000). Among women, Brazil recorded 132,133 deaths, whereas Jamaica had the highest ASMR (110.6 per 100,000). Prostate cancer was the leading cause of cancer death among men (60,792 deaths; ASMR = 13.9 per 100,000), and breast cancer was the primary cause among women (61,228 deaths; ASMR = 13.2 per 100,000), underscoring its significant burden across LAC (Fig. 3; Table 2).

The relationship between HDI and cancer indicators in LAC showed contrasting patterns (Fig. 3). Linear regression revealed a significant inverse association between HDI and MIR [β = −0.66; R^2^ = 0.49; 95% confidence interval (CI) for r = 0.46–0.85; *P* = 1.6 × 10^−5^], indicating improved survival outcomes in more developed countries. Conversely, HDI was positively associated with ASMR (β = 344.8; R^2^ = 0.43; 95% CI for r = 0.15–0.72; *P* = 7.51 × 10^−5^), reflecting higher mortality counts likely due to enhanced detection and reporting in higher-HDI settings. The association between HDI and ASIR was not statistically significant (β = 66.2; R^2^ = 0.09; 95% CI for r = 0.00–0.59; *P* = 0.115; Supplementary Table S5).

In 2022, the average MIR in LAC was 0.52 in countries with very high HDI, 0.50 in high-HDI countries, and 0.59 in countries with a medium HDI. Among countries with very high HDI, MIRs ranged from 0.40 in French Guiana to 0.53 in Argentina and Uruguay. Haiti and Honduras recorded the highest values at 0.65, followed by Bolivia (0.63), Jamaica (0.62), and Nicaragua (0.57). These data reflect variation in cancer outcomes across the region, with MIR values ranging from 0.40 to 0.66.

### Early-onset cancers

In 2022, early-onset cancers (diagnosed between ages 15 and 50 years) accounted for 269,881 cases and 85,695 deaths in LAC. Brazil reported the highest number of early-onset cancer cases (99,616 cases) and deaths (30,036 deaths), followed by Mexico (48,169 cases and 14,649 deaths) and Argentina (22,434 cases and 6,417 deaths; see Supplementary Table S1; Supplementary Fig. S1). Among men and women, the highest ASIR for early-onset cancers was observed in Uruguay (75.5 per 100,000 and 168.9 per 100,000, respectively). The most common early-onset cancers among women were breast cancer (68,971 cases; ASIR = 36.9 per 100,000) and cervical cancer (26,635 cases; ASIR = 14.2 per 100,000). Among men, colorectal cancer (7,962 cases; ASIR = 4.6 per 100,000) and testicular cancer (10,847 cases; ASIR = 6.2 per 100,000) were the most frequently diagnosed (see Supplementary Table S2; Supplementary Fig. S2).

Early-onset colorectal cancer mortality (Supplementary Table S8) increased significantly among men in Chile (APC, 95% CI, 2.52; 1.96–3.08), Colombia (1.72; 1.20–2.26), Costa Rica (1.72; 0.71–2.74), Guatemala (2.63; 1.30–3.98), Mexico (2.56; 2.24–2.89), Paraguay (4.45; 2.59–6.30), and Brazil (1.85; 1.70–2.00). Among women, mortality increased in Chile (2.11; 1.48–2.75), Costa Rica (2.63; 1.72–3.55), Guatemala (2.17; 1.45–2.89), Mexico (1.88; 1.59–2.16), and Paraguay (5.94; 3.72–8.29).

Early-onset lung cancer mortality declined sharply in Chile (APC, −3.96; 95% CI, −4.58 to −3.32), Colombia (−2.93; −3.48 to −2.37), Costa Rica (−2.36; −3.75 to −0.92), Cuba (−3.63; −4.13 to −3.13), Argentina (−5.45; −5.72 to −5.18), Brazil (−2.57; −2.84 to −2.29), and Uruguay (−3.89; −4.74 to −3.02). Among women, decreasing trends were also evident in Chile (−1.45; −1.99 to −0.91), Colombia (−1.73; −2.20 to −1.26), Cuba (−2.78; −3.55 to −1.99), and Argentina (−1.59; −2.05 to −1.12), whereas other countries showed stable or modestly increasing rates. Additionally, in the 15- to 39-year subgroup, breast (women) and testicular (men) cancers were the most incident; thyroid and cervix uteri (women) and leukemia and colorectum (men) followed. Despite lower incidence, cervical cancer accounted for the highest female mortality, whereas leukemia and brain/central nervous system led male mortality (Supplementary Fig. S6).

By 2050, early-onset cancer incidence is projected to increase in lower-HDI countries such as Haiti, Guatemala, and Honduras while remaining relatively stable in higher-HDI countries (Supplementary Table S3; Supplementary Fig. S5). The most significant proportional increases are projected for Haiti, Bolivia, and Guatemala. The number of early-onset cancer cases in LAC is projected to increase from approximately 269,881 cases in 2022 to more than 283,758 cases, representing a net increase of more than 26,000 new cases (+5%).

### Past and future trends

**Figure 4.**
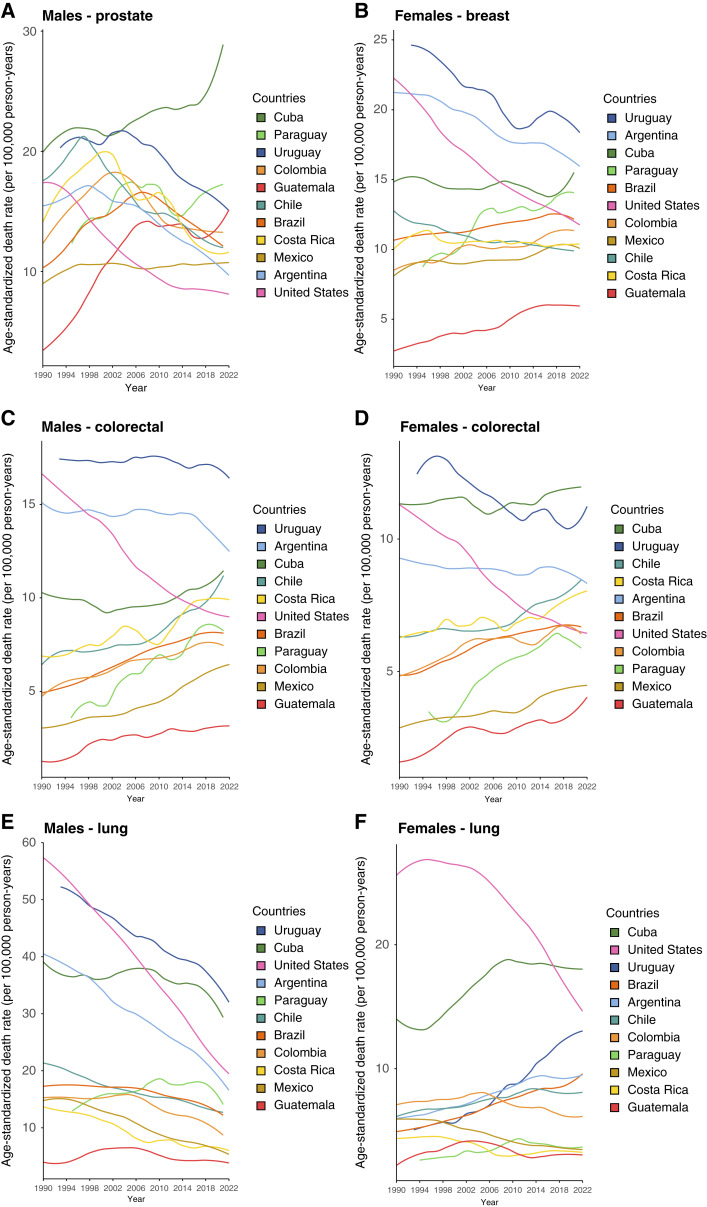
ASMR in LAC countries by cancer type, 1990–2022. **A,** Prostate cancer in males. **B,** Breast cancer in females. **C,** Colorectal cancer in males. **D,** Colorectal cancer in females. **E,** Lung cancer in males. **F,** Lung cancer in females.

Between 1990 and 2022, cancer mortality trends in LAC varied considerably by country, sex, and cancer type (Fig. 4).

Prostate cancer mortality (Table 3) declined significantly in Argentina (APC, −1.52; 95% CI, −1.80 to −1.24), Chile (−1.71; −2.01 to −1.40), and Uruguay (−1.13; −1.43 to −0.82), remained stable in Colombia (−0.42; −0.89 to 0.04) and Costa Rica (−1.36; −1.88 to −0.85), and increased in Guatemala (+3.91; 2.93–4.91) and Mexico (+0.26; 0.09–0.42). In contrast, the United States showed a steeper decline (−2.72; −2.94 to −2.51). Breast cancer mortality among women declined in Uruguay (−1.02; −1.27 to −0.77) and Argentina (−0.92; −1.01 to −0.83), stabilized in Chile (−0.72; −0.85 to −0.59) and Costa Rica (−0.13; −0.42 to 0.16), and increased in Brazil (+0.53; 0.46–0.59), Mexico (+0.55; 0.43–0.67), and Guatemala (+2.63; 2.05–3.21).

**Table 3.** APC in cancer mortality (1990–2022) for top three most incident cancer types.

Country	Male			Female		
	Prostate	Colorectal	Lung	Breast	Colorectal	Lung
Chile	**−1.71 (−2.01 to −1.40)**	**1.44 (1.18–1.70)**	**−1.61 (−1.76 to −1.45)**	**−0.72 (−0.85 to −0.59)**	**0.89 (0.71–1.06)**	**1.04 (0.86–1.22)**
Colombia	−0.42 (−0.89 to 0.04)	**1.41 (1.24–1.57)**	**−1.49 (−1.84 to −1.15)**	**0.86 (0.69–1.03)**	**0.96 (0.75–1.17)**	**−0.64 (−0.97 to −0.31)**
Costa Rica	**−1.36 (−1.88 to −0.85)**	**1.27 (0.88–1.66)**	**−2.75 (−3.19 to −2.29)**	−0.13 (−0.42 to 0.16)	**0.63 (0.29–0.97)**	**−1.61 (−2.09 to −1.13)**
Cuba	**0.73 (0.55–0.91)**	**0.28 (0.04–0.52)**	**−0.43 (−0.62 to −0.25)**	−0.15 (−0.31 to 0.00)	0.13 (0.00–0.26)	**1.21 (0.94–1.47)**
Argentina	**−1.52 (−1.80 to −1.24)**	**−0.31 (−0.44 to −0.18)**	**−2.50 (−2.68 to −2.33)**	**−0.92 (−1.01 to −0.83)**	**−0.19 (−0.29 to −0.09)**	**1.51 (1.32–1.70)**
Guatemala	**3.91 (2.93–4.91)**	**3.00 (2.22–3.79)**	−0.14 (−1.01 to 0.74)	**2.63 (2.05–3.21)**	**2.34 (1.80–2.88)**	0.00 (−0.70 to 0.71)
Mexico	**0.26 (0.09–0.42)**	**2.48 (2.30–2.67)**	**−3.27 (−3.49 to −3.05)**	**0.55 (0.43–0.67)**	**1.34 (1.16–1.52)**	**−1.90 (−2.04 to −1.75)**
Paraguay	**0.66 (0.11–1.21)**	**3.33 (2.71–3.96)**	**0.74 (0.23–1.26)**	**1.76 (1.32–2.20)**	**2.97 (2.33–3.62)**	**1.31 (0.46–2.17)**
Brazil	**0.53 (0.03–1.03)**	**1.82 (1.69–1.95)**	**−0.95 (−1.13 to −0.77)**	**0.53 (0.46–0.59)**	**1.21 (1.08–1.34)**	**1.88 (1.71–2.05)**
United States of America	**−2.72 (−2.94 to −2.51)**	**−2.12 (−2.21 to −2.02)**	**−3.27 (−3.49 to −3.05)**	**−1.99 (−2.06 to −1.92)**	**−1.94 (−2.03 to −1.84)**	**−1.63 (−1.95 to −1.31)**
Uruguay	**−1.13 (−1.43 to −0.82)**	−0.10 (−0.31 to 0.12)	**−1.48 (−1.65 to −1.31)**	**−1.02 (−1.27 to −0.77)**	**−0.82 (−1.01 to −0.62)**	**3.61 (3.16–4.08)**

Values represent APC and 95% CI in ASMRs (1990–2022). Bold indicates statistical significance (*P* < 0.05).

Colorectal cancer mortality increased in most LAC countries, especially among men in Mexico (+2.48; 2.30–2.67) and Paraguay (+3.33; 2.71–3.96), whereas Argentina (−0.31; −0.44 to −0.18) and Uruguay (−0.10; −0.31 to 0.12) showed mild decline or stabilization. Among women, increases were noted in Mexico (+1.34; 1.16–1.52) and Paraguay (+2.97; 2.33–3.62), whereas Uruguay (−0.82; −1.01 to −0.62) showed a modest decline. By comparison, the United States reported consistent decreases (men, −2.12; −2.21 to −2.02; women, −1.94; −2.03 to −1.84).

Lung cancer mortality declined significantly among men in Chile (−1.61; −1.76 to −1.45), Argentina (−2.50; −2.68 to −2.33), and Uruguay (−1.48; −1.65 to −1.31), while remaining stable in Costa Rica and Mexico. Among women, mortality increased in nearly all countries, more than doubling in Uruguay (+3.61; 3.16–4.08) and increasing in Argentina (+1.51; 1.32–1.70), Brazil (+1.88; 1.71–2.05), and Chile (+1.04; 0.86–1.22).

Projections for 2050 indicate that the annual number of new cancer cases in LAC will reach approximately 2.9 million, with over 1.5 million cancer-related deaths (Table 4). The total number of new cancer cases is expected to increase from 768,843 in 2022 to 1,506,210 in 2050 among males (a 95.9% increase) and from 782,217 to 1,365,053 among females (a 74.5% increase). Brazil is expected to experience the most significant absolute increases for both sexes. Among men, the incidence is projected to nearly double from 319,711 to 626,225 (96% increase), whereas mortality will increase from 146,702 to 301,873 (106% increase). Among women in Brazil, the incidence is anticipated to grow by 71% (from 307,482–524,722) and mortality by 91% (from 132,133–251,938). Several smaller countries, such as Honduras and The Bahamas, are expected to witness a doubling in incidence and mortality for certain sex-specific groups, reflecting significant proportional increases from lower baseline values. The projected incidence and mortality with regard to each cancer site are displayed on Supplementary Table S6.

**Table 4.** Estimated incident cancer cases and deaths for 2022 and projected values for 2050, stratified by country and sex across all ages.

Region	Male incidence		Female incidence		Male mortality		Female mortality	
	2022 estimated incidence	2050 projected incidence	2022 estimated incidence	2050 projected incidence	2022 estimated mortality	2050 projected mortality	2022 estimated mortality	2050 projected mortality
Argentina	65,040	117,193	68,380	102,482	36,588	69,828	33,663	54,855
The Bahamas	477	984	478	975	266	656	286	672
Barbados	578	677	542	639	381	493	357	457
Bolivia	7,692	12,384	9,887	17,196	4,958	7,546	6,057	10,408
Brazil	319,711	626,225	307,482	524,722	146,702	301,873	132,133	251,938
Belize	188	458	221	529	106	273	105	284
Chile	32,800	69,969	27,076	46,522	16,897	40,016	14,543	28,102
Colombia	56,224	117,160	61,396	111,192	27,816	61,915	28,903	60,023
Costa Rica	6,808	14,503	6,517	11,901	3,246	7,448	2,826	6,082
Cuba	26,892	36,009	22,796	29,520	15,854	24,053	11,870	17,374
Dominican Republic	10,735	20,421	9,436	17,020	6,176	12,915	5,568	11,123
Ecuador	13,890	32,329	16,998	35,275	7,658	19,022	8,500	19,787
El Salvador	4,205	6,584	5,594	9,013	2,290	3,632	3,002	5,240
French Guyana	341	841	248	577	138	447	95	259
France, Guadeloupe	1,312	1,201	811	965	533	689	419	612
Guatemala	7,783	18,770	10,018	22,290	4,544	10,908	5,825	13,738
Guyana	537	801	688	1,050	271	410	341	552
Haiti	7,028	12,143	6,832	12,155	4,595	7,918	4,419	7,988
Honduras	5,051	12,258	5,764	12,393	3,364	7,933	3,647	8,475
Jamaica	3,713	5,352	3,787	5,397	2,423	3,487	2,209	3,410
France, Martinique	1,175	1,152	882	965	459	552	427	592
Mexico	95,954	193,239	111,200	199,645	46,415	101,408	49,795	102,953
Nicaragua	3,763	9,062	4,646	9,810	2,306	5,649	2,516	5,640
Panama	4,156	9,123	4,197	8,082	1,963	4,623	1,807	3,923
Paraguay	6,966	12,498	6,817	12,390	3,532	6,594	3,049	6,152
Peru	33,724	66,645	39,103	72,588	16,790	34,456	19,144	38,702
Puerto Rico	7,486	9,789	6,292	7,885	3,212	4,878	2,584	3,850
Saint Lucia	265	392	183	274	132	197	91	159
Suriname	571	1,124	548	972	340	722	286	582
Trinidad and Tobago	1,969	3,316	1,962	3,182	1,169	2,306	1,052	1,993
Uruguay	8,579	12,441	8,238	10,209	4,920	7,583	4,060	5,296
Venezuela	31,374	55,950	31,573	55,597	16,346	30,434	15,391	29,003

Under a constant-rate scenario (0% annual change), new cancer cases will increase from 768,000 to 1,118,000 among males and from 781,000 to 1,014,000 among females by 2050 (Supplementary Fig. S7). A sustained 2% annual decrease in mortality could prevent approximately 916,000 cancer deaths in males and 821,000 in females by 2050, compared with a +2% growth scenario.

## Discussion

This updated assessment of the cancer burden in LAC, based on GLOBOCAN 2022 data, reveals emerging challenges with early-onset cancers and widening disparities in outcomes caused by socioeconomic inequities and epidemiologic transitions. In 2022, the region recorded 1.55 million new cancer cases and 749,000 cancer-related deaths. Prostate and breast cancers were the most frequently diagnosed malignancies among men and women, respectively, whereas lung and breast cancers accounted for the highest mortality rates. Trends in cancer mortality varied by sex, showing a decline in lung cancer mortality among men but increasing rates among women. Projections indicate a substantial increase in the cancer burden by 2050 as observed globally.

Building upon previous GLOBOCAN 2020 estimates, this study offers a more contemporary evaluation of cancer epidemiology in LAC. The 2020 study estimated 1.5 million new cancer cases and 700,000 deaths, with an incidence rate of 186.5 per 100,000 and a mortality rate of 86.6 per 100,000 (16). Although the overall figures remain comparable, our updated data reflect shifts in ASIR and ASMR, particularly in infection- and lifestyle-related cancers. The ongoing decline in mortality from infection-driven cancers, such as cervical and stomach cancers, parallels the increasing incidence of colorectal and breast cancers, aligning with broader epidemiologic transitions in LAC (6). Additionally, our study highlights emerging patterns of early-onset malignancies, an area not previously explored in detail.

Marked disparities in cancer incidence and mortality persist across LAC, reflecting variations in healthcare infrastructure, screening coverage, and treatment availability (17). In our analysis, there was a significant inverse correlation between HDI and MIR (*P* = 0.008), suggesting better outcomes in more developed countries. For example, breast cancer mortality remains disproportionately high in lower-HDI nations, underscoring gaps in screening programs and access to systemic therapies (18). Similarly, cervical cancer, largely preventable through human papillomavirus (HPV) vaccination and screening, remains a leading cause of death in women from low-resource countries, emphasizing the need for targeted interventions to improve primary and secondary prevention (19).

Distinct sex-specific trends highlight changing cancer patterns. Among men, prostate cancer remains the most diagnosed malignancy, but lung cancer is the leading cause of mortality, reinforcing the long-term impact of historical smoking patterns (20). Notably, countries such as Argentina, Chile, and Uruguay, which have implemented comprehensive tobacco control policies, have experienced significant declines in male lung cancer mortality, with APC values of −2.50, −1.61, and −1.48, respectively (21, 22). In contrast, lung cancer mortality in women continues to increase, particularly in higher-HDI countries. This finding may be partly explained by the increase in tobacco use in this population. However, there could also be a greater predisposition due to ancestry as recent studies suggest that exposure to tiny particles in the air, or fine particular matter (PM2.5), may be responsible for some mutations in nonsmoking women (23, 24). In addition, a predisposition to hormone fluctuations may influence tumor growth in women. Estrogen receptors are found in lung tissue, and experimental studies suggest that estrogen promotes tumor growth (25, 26).

Colorectal cancer incidence and mortality have increased in both sexes. Once considered a disease of older adults, colorectal cancer is now increasingly diagnosed in individuals under 50 years of age. This shift likely reflects changes in diet, escalating obesity rates, and low adherence to screening guidelines and suggests an urgent need to reevaluate age thresholds for screening (27). Dietary transitions toward ultra-processed foods (UPF), sedentary lifestyles, and increasing metabolic disease further exacerbate this risk (28). A World Health Organization/Pan American Health Organization study across 13 LAC countries identified a strong correlation (R^2^ = 0.76) between UPF consumption and obesity prevalence (29). Brazil, for example, has witnessed parallel increases in UPF intake, obesity, diabetes, and cardiometabolic mortality. Simultaneously, poor adherence to screening protocols delays early detection, reducing the likelihood of curative treatment (30). This convergence of risk factors calls for comprehensive public health interventions, including improved nutrition, physical activity, and expanded access to colorectal cancer screening.

Although prostate and breast cancers were the most diagnosed cancers across nearly all countries in LAC, Bolivia stood out with cervical cancer as the leading malignancy among women. This pattern may reflect differences in the implementation and effectiveness of screening programs and HPV vaccination (31, 32). Indeed, recent analyses show that only 42% of women aged 25 to 65 years in Bolivia were screened for cervical cancer within the previous 3 years, which is below the 70% threshold recommended by the World Health Organization. Across Latin America, more than two thirds of countries also fall short of this benchmark. Cytology remains the most common screening modality, and HPV testing is not yet widely implemented, particularly in low-resource settings (31). These disparities in screening coverage highlight the need for stronger surveillance systems and broader adoption of HPV testing to meet regional elimination targets.

Shifts in the burden of liver cancer in Central America further illustrate the regional variation in cancer patterns. In Central America, liver cancer is a leading cause of cancer death in countries such as Guatemala and Honduras, surpassing lung and prostate cancers. This likely reflects a high burden of chronic liver disease, fueled by hepatitis B and C infections and metabolic dysfunction–associated steatotic liver disease. In Guatemala, hepatitis B virus and hepatitis C virus prevalence rates exceed 1,000 and 3,000 per 100,000, respectively, whereas metabolic dysfunction–associated steatotic liver disease affects more than 18,000 per 100,000 (33). Additionally, exposure to aflatoxin B1, a hepatocarcinogen, has been implicated as a contributing risk factor for chronic liver disease in Guatemala (34). These patterns underscore the need for comprehensive national strategies encompassing viral hepatitis control, liver disease prevention, and environmental toxin mitigation.

Meanwhile, the burden of early-onset cancers in LAC is significant and increasing, particularly in Brazil and Mexico, where the highest absolute number of cases and deaths were observed. Breast cancer is the most common early-onset malignancy in women, whereas in men, colorectal cancer and testicular cancer are predominant. Increasing early-onset colorectal cancer mortality, particularly in Brazil, Mexico, and Argentina, underscores the need for earlier detection strategies and increased awareness of symptoms among younger populations. By 2050, early-onset cancer incidence in LAC is projected to grow by 5%, from 270,000 to more than 283,000 cases, with the most significant proportional increases expected in low-HDI countries such as Haiti, Guatemala, and Bolivia. Public health efforts should prioritize awareness campaigns, symptom recognition (35), and improved access to screening and early intervention services for this population.

Addressing the growing cancer burden also requires confronting systemic barriers to innovation and equitable access to treatment modalities and genetic testing across the region (36). Adoption of precision oncology strategies, including biomarker testing, targeted therapies, and immunotherapy, has been slow and uneven, restricted largely to major urban centers and private sectors (37). In addition, participation in clinical trials remains extremely limited across most LAC countries, with activity concentrated in a few urban academic centers and predominantly within private healthcare systems. This limits not only patient access to innovative therapies but also the generation of region-specific evidence and inclusion in global research efforts. Regulatory hurdles, insufficient research infrastructure, and a shortage of trained clinical researchers further exacerbate this disparity (36).

Beyond access to novel therapies, profound inequities exist in the availability of evidence-based, essential cancer medicines. According to a global survey of oncologists across 82 countries, the proportion of respondents reporting universal availability of the 20 most important cancer medicines was as low as 9% to 54% in low- and lower-middle-income countries (38). In several parts of LAC, even access to basic, life-saving treatments remains inconsistent, particularly in public health systems in which stockouts and supply chain disruptions are common (39). A significant driver of survival disparities is the limited availability of affordable, quality-assured cancer treatments. Although very high HDI countries report MIRs ranging from 0.40 to 0.53, medium-HDI countries such as Haiti, Honduras, Bolivia, and Nicaragua exhibit substantially higher values (0.57–0.65). Therefore, improving the outcomes of patients with cancer in LAC requires urgent and coordinated efforts to strengthen cancer research networks, secure consistent access to essential medicines and diagnostics, and streamline regulatory pathways, transforming cancer care in LAC from a fragmented system of privilege into one that delivers equitable and life-saving care for all.

One of the major limitations of this study lies in the analysis of population-based projections derived from national or population-based cancer registries (40). There is an apparent inequity in the coverage of cancer registries worldwide: high-quality cancer registries cover only 4%, 8%, and 7% of the populations in Africa, Asia, and Latin America, respectively, whereas the equivalent coverage is 83% in North America and 32% in Europe (40, 41). Another limitation is the predominance of cancer epidemiology studies published in Spanish and Portuguese, which restricts their accessibility in major international databases and limits dissemination compared with countries with very high HDI scores (42, 43). Furthermore, although cancer remains a major cause of premature death, it has increasingly become a chronic condition for certain types, particularly in countries approaching very high human development. This shift, driven by earlier diagnosis and improved treatment, highlights the need for registries to report not only incidence and mortality but also survival rates, years of life lost, and years lived with disability after diagnosis (44). Although our analysis does not permit quantification of the proportion of health budgets devoted to cancer prevention, screening, and treatment, a recent regional health financing review highlights large disparities in preventive versus curative care spending across Latin American countries (45).

Additionally, although we modeled seven hypothetical scenarios assessing the potential impacts of annual increases or decreases (0%, ±1%, ±2%, and ±3%) in age-specific incidence and mortality rates, these simulations are speculative and should be interpreted with caution. Given that projections were based on constant-rate demographic assumptions, modest percentage changes are likely within the range of demographic uncertainty and should not be interpreted as meaningful increases. Finally, although the MIR serves as a useful proxy for survival, it remains an imperfect surrogate for capturing differences in stage at diagnosis, access to care, and treatment quality across countries (46).

### Conclusion

Our findings underscore the escalating cancer burden in LAC. Although mortality has declined for certain cancers, the continued increase in colorectal, breast, and early-onset cancers signals a growing public health crisis that demands immediate attention. As the region undergoes demographic and epidemiologic transitions, the need for sustained and equity-driven investments in cancer surveillance, prevention, and early detection is critical. Failure to act will result in an overwhelming strain on already fragile health systems. A coordinated regional response that prioritizes health system strengthening, equitable access to essential medicines and diagnostics, and the expansion of cancer research infrastructure is imperative to alter the current trajectory and improve outcomes for future generations.

## Supplementary Material

Supplementary Table S1 Absolute counts of early-onset cancer cases and deaths, age-standardized incidence (ASIR) and mortality (ASMR) rates, and Human Development Index (HDI) values, stratified by country and gender, 2022.

Supplementary Table S2 Table S2. Absolute counts of early-onset cases and deaths with corresponding age-standardized incidence (ASIR) and mortality (ASMR) rates, stratified by cancer type and gender, 2022.

Supplementary Table S3 Table S3. Estimated incident early-onset cancer cases and deaths for 2022 and projected values for 2050, stratified by country and sex.

Supplementary Table S4 Table S4. Annual Percent Change (APC) in Cancer Mortality (1990–2022) for top 3 most incident early-onset cancers.

Supplementary Table S5 Table S5. Linear Regression Between Human Development Index (HDI) and Cancer Burden Indicators (1990–2022)

Supplementary Table S6 Table S6. Estimated incident cancer cases and deaths for 2022 and projected values for 2050, stratified by cancer type and sex.

Supplementary Figure S1 Figure S1. ASIR and ASMR in 2022 for all countries in patients with early-onset cancer.

Supplementary Figure S2 Figure S2. Cancer types by ASIR and ASMR in 2022 in patients with early-onset cancer.

Supplementary Figure S3 Figure S3. Projected cases and deaths numbers (per 1,000 persons) under varying global rate-change scenarios in patients with early-onset cancer, 2022–2050.

Supplementary Figure S4 Figure S4. ASMR in LAC countries by age group and sex. All sites excluding non-melanoma skin cancer, 1990–2022.

Supplementary Figure S5 Figure S5. ASMR in LAC countries by cancer type in patients with early-onset cancer, 1990–2022.

Supplementary Figure S6 Figure S6. Bar plots of ASIR and ASMR in 2022 for all countries and cancer types in patients with 15-39 years.

Supplementary Figure S7 Figure S7. Projected cases and deaths numbers (per 1,000 persons) under varying global rate-change scenarios, 2022–2050.

## Data Availability

All data are publicly available through the Global Cancer Observatory and UN World Population Prospects websites. No additional unpublished data are available.
