# Supplementary material for: Cancer Incidence and Mortality Estimates in Latin America and the Caribbean: A Systematic Analysis of the GLOBOCAN 2022
Source: Cancer Res Commun. 2025 Dec 29;5(12):2236–48. doi: 10.1158/2767-9764.CRC-25-0564 (PMC12745351; doi:10.1158/2767-9764.CRC-25-0564)
Supplement: Supplementary Table S1 — Absolute counts of early-onset cancer cases and deaths, age-standardized incidence (ASIR) and mortality (ASMR) rates, and Human Development Index (HDI) values, stratified by country and gender, 2022. [file crc-25-0564_supplementary_table_s1_suppst1.docx]

**Supplementary Table 1.** Absolute counts of early-onset cancer cases and deaths, age-standardized incidence (ASIR) and mortality (ASMR) rates, and Human Development Index (HDI) values, stratified by country and sex, 2022.

| **Country** | **Male** | | | |  | **Female** | | | |  | **Total** | |  | **HDI** |
| --- | --- | --- | --- | --- | --- | --- | --- | --- | --- | --- | --- | --- | --- | --- |
|  | Absolute Incidence | ASIR | Absolute mortality | ASMR |  | Absolute Incidence | ASIR | Absolute mortality | ASMR |  | Absolute incidence | Absolute mortality |  | Value |
| Argentina | 7017 | 59.7 | 2472 | 21.0 |  | 15417 | 125.6 | 3945 | 32.0 |  | 22434 | 6417 |  | 0.849 |
| Bahamas | 58 | 54.2 | 11 | 10.0 |  | 109 | 93.1 | 37 | 30.9 |  | 167 | 48 |  | 0.82 |
| Barbados | 49 | 65.6 | 11 | 14.3 |  | 83 | 107.5 | 24 | 30.0 |  | 132 | 35 |  | 0.809 |
| Bolivia | 935 | 32.6 | 515 | 18.0 |  | 3278 | 112.0 | 1173 | 41.3 |  | 4213 | 1688 |  | 0.698 |
| Brazil | 30630 | 51.1 | 12334 | 20.5 |  | 68986 | 109.4 | 17702 | 28.0 |  | 99616 | 30036 |  | 0.76 |
| Belize | 16 | 14.8 | 4 | 3.5 |  | 71 | 65.9 | 25 | 23.0 |  | 87 | 29 |  | 0.7 |
| Chile | 2371 | 45.6 | 839 | 16.0 |  | 4262 | 80.5 | 1126 | 21.2 |  | 6633 | 1965 |  | 0.86 |
| Colombia | 6452 | 47.1 | 2633 | 19.4 |  | 14518 | 100.2 | 3950 | 27.5 |  | 20970 | 6583 |  | 0.758 |
| Costa Rica | 647 | 45.9 | 241 | 17.0 |  | 1415 | 98.5 | 335 | 23.5 |  | 2062 | 576 |  | 0.806 |
| Cuba | 1515 | 52.5 | 564 | 19.2 |  | 2560 | 91.5 | 692 | 24.2 |  | 4075 | 1256 |  | 0.764 |
| Dominican Republic | 1519 | 54.4 | 609 | 21.8 |  | 2994 | 103.8 | 1099 | 38.7 |  | 4513 | 1708 |  | 0.766 |
| Ecuador | 1692 | 36.2 | 767 | 16.5 |  | 4241 | 89.8 | 1264 | 27.0 |  | 5933 | 2031 |  | 0.765 |
| El Salvador | 599 | 40.2 | 287 | 19.5 |  | 1381 | 75.9 | 474 | 26.2 |  | 1980 | 761 |  | 0.674 |
| French Guyana | 30 | 39.3 | 6 | 7.8 |  | 63 | 78.4 | 11 | 13.6 |  | 93 | 17 |  | 0.79 |
| France, Guadeloupe | 59 | 76.8 | 20 | 25.2 |  | 103 | 105.5 | 23 | 22.6 |  | 162 | 43 |  | 0.86 |
| Guatemala | 1205 | 27.3 | 664 | 15.3 |  | 3064 | 66.7 | 1190 | 26.7 |  | 4269 | 1854 |  | 0.629 |
| Guyana | 43 | 22.4 | 27 | 14.0 |  | 163 | 85.8 | 58 | 30.8 |  | 206 | 85 |  | 0.742 |
| Haiti | 1177 | 42.9 | 679 | 25.7 |  | 2041 | 68.5 | 1003 | 35.1 |  | 3218 | 1682 |  | 0.552 |
| Honduras | 951 | 36.2 | 674 | 25.5 |  | 2136 | 81.7 | 971 | 37.6 |  | 3087 | 1645 |  | 0.624 |
| Jamaica | 357 | 46.7 | 151 | 20.0 |  | 1004 | 122.8 | 390 | 48.0 |  | 1361 | 541 |  | 0.706 |
| France, Martinique | 21 | 29.1 | 10 | 13.1 |  | 97 | 108.3 | 21 | 22.1 |  | 118 | 31 |  | 0.854 |
| Mexico | 15760 | 46.3 | 5920 | 17.5 |  | 32409 | 87.8 | 8729 | 23.7 |  | 48169 | 14649 |  | 0.781 |
| Nicaragua | 660 | 37.1 | 374 | 21.4 |  | 1314 | 70.6 | 538 | 29.0 |  | 1974 | 912 |  | 0.669 |
| Panama | 455 | 39.3 | 160 | 13.9 |  | 1148 | 98.2 | 291 | 24.8 |  | 1603 | 451 |  | 0.82 |
| Paraguay | 961 | 51.5 | 333 | 18.3 |  | 2283 | 124.4 | 614 | 34.8 |  | 3244 | 947 |  | 0.731 |
| Peru | 4017 | 43.8 | 1715 | 18.8 |  | 9693 | 104.7 | 2905 | 31.6 |  | 13710 | 4620 |  | 0.762 |
| Puerto Rico | 510 | 74.7 | 125 | 17.7 |  | 926 | 127.7 | 140 | 18.2 |  | 1436 | 265 |  | N/A |
| Saint Lucia | 19 | 37.7 | 2 | 4.1 |  | 55 | 97.9 | 11 | 19.8 |  | 74 | 13 |  | 0.725 |
| Suriname | 73 | 48.2 | 28 | 18.6 |  | 160 | 103.0 | 37 | 24.2 |  | 233 | 65 |  | 0.69 |
| Trinidad and Tobago | 224 | 57.6 | 60 | 16.2 |  | 457 | 111.7 | 127 | 31.3 |  | 681 | 187 |  | 0.814 |
| Uruguay | 677 | 75.5 | 199 | 22.0 |  | 1281 | 139.8 | 278 | 30.0 |  | 1958 | 477 |  | 0.83 |
| Venezuela | 3645 | 49.5 | 1454 | 19.9 |  | 7429 | 94.8 | 2498 | 31.9 |  | 11074 | 3952 |  | 0.699 |
