# Supplementary material for: Cancer Incidence and Mortality Estimates in Latin America and the Caribbean: A Systematic Analysis of the GLOBOCAN 2022
Source: Cancer Res Commun. 2025 Dec 29;5(12):2236–48. doi: 10.1158/2767-9764.CRC-25-0564 (PMC12745351; doi:10.1158/2767-9764.CRC-25-0564)
Supplement: Supplementary Table S2 — Table S2. Absolute counts of early-onset cases and deaths with corresponding age-standardized incidence (ASIR) and mortality (ASMR) rates, stratified by cancer type and gender, 2022. [file crc-25-0564_supplementary_table_s2_suppst2.docx]

**Supplementary Table 2.** Absolute counts of early-onset cases and deaths with corresponding age-standardized incidence (ASIR) and mortality (ASMR) rates, stratified by cancer type and sex, 2022.

| **Cancer type** | **ICD** | **Male** | | | |  | **Female** | | | |  | **Total** | |
| --- | --- | --- | --- | --- | --- | --- | --- | --- | --- | --- | --- | --- | --- |
|  |  | Absolute Incidence | ASIR | Absolute mortality | ASMR |  | Absolute Incidence | ASIR | Absolute mortality | ASMR |  | Absolute incidence | Absolute mortality |
| Lip, oral cavity | C00-06 | 2241 | 1.3 | 663 | 0.39 |  | 949 | 0.52 | 242 | 0.13 |  | 3190 | 905 |
| Salivary glands | C07-08 | 544 | 0.31 | 81 | 0.05 |  | 556 | 0.30 | 60 | 0.03 |  | 1100 | 141 |
| Oropharynx | C09-10 | 1203 | 0.71 | 452 | 0.27 |  | 271 | 0.15 | 80 | 0.04 |  | 1474 | 532 |
| Nasopharynx | C11 | 387 | 0.23 | 164 | 0.10 |  | 174 | 0.10 | 72 | 0.04 |  | 561 | 236 |
| Hypopharynx | C12-13 | 184 | 0.11 | 68 | 0.04 |  | 52 | 0.03 | 12 | 0.01 |  | 236 | 80 |
| Oesophagus | C15 | 1418 | 0.83 | 1146 | 0.67 |  | 346 | 0.19 | 237 | 0.13 |  | 1764 | 1383 |
| Stomach | C16 | 4005 | 2.3 | 3174 | 1.8 |  | 3822 | 2.1 | 2936 | 1.6 |  | 7827 | 6110 |
| Colorectum | C18-C21 | 7962 | 4.6 | 3310 | 1.9 |  | 8664 | 4.7 | 3375 | 1.8 |  | 16626 | 6685 |
| Liver and intrahepatic bile ducts | C22 | 1526 | 0.88 | 1459 | 0.84 |  | 1434 | 0.79 | 1290 | 0.70 |  | 2960 | 2749 |
| Gallbladder | C23 | 203 | 0.12 | 112 | 0.06 |  | 630 | 0.34 | 348 | 0.19 |  | 833 | 460 |
| Pancreas | C25 | 1559 | 0.89 | 1253 | 0.72 |  | 1245 | 0.68 | 961 | 0.53 |  | 2804 | 2214 |
| Larynx | C32 | 1115 | 0.65 | 588 | 0.35 |  | 305 | 0.17 | 101 | 0.05 |  | 1420 | 689 |
| Trachea, bronchus and lung | C33-34 | 2718 | 1.6 | 2012 | 1.1 |  | 2630 | 1.5 | 2031 | 1.1 |  | 5348 | 4043 |
| Melanoma of skin | C43 | 2089 | 1.2 | 437 | 0.25 |  | 2211 | 1.2 | 366 | 0.20 |  | 4300 | 803 |
| Non-melanoma skin cancer | C44 | 2410 | 1.4 | 261 | 0.15 |  | 1843 | 1 | 172 | 0.09 |  | 4253 | 433 |
| Mesothelioma | C45 | 60 | 0.03 | 47 | 0.03 |  | 48 | 0.03 | 39 | 0.02 |  | 108 | 86 |
| Kaposi sarcoma | C46 | 1483 | 0.82 | 291 | 0.16 |  | 199 | 0.11 | 36 | 0.02 |  | 1682 | 327 |
| Breast | C50 | - | - | - | - |  | 68971 | 36.9 | 12652 | 6.8 |  | 68971 | 12652 |
| Vulva | C51 | - | - | - | - |  | 426 | 0.23 | 75 | 0.04 |  | 426 | 75 |
| Vagina | C52 | - | - | - | - |  | 279 | 0.15 | 62 | 0.03 |  | 279 | 62 |

| Cervix uteri | C53 | - | - | - | - |  | 26635 | 14.2 | 10062 | 5.4 |  | 26635 | 10062 |
| --- | --- | --- | --- | --- | --- | --- | --- | --- | --- | --- | --- | --- | --- |
| Corpus uteri | C54 | - | - | - | - |  | 4929 | 2.7 | 646 | 0.35 |  | 4929 | 646 |
| Ovary | C56 | - | - | - | - |  | 7041 | 3.8 | 2970 | 1.6 |  | 7041 | 2970 |
| Penis | C60 | 845 | 0.48 | 282 | 0.16 |  | - | - | - | - |  | 845 | 282 |

| Prostate | C61 | 3727 | 2.1 | 463 | 0.27 |  | - | - | - | - |  | 3727 | 463 |
| --- | --- | --- | --- | --- | --- | --- | --- | --- | --- | --- | --- | --- | --- |
| Testis | C62 | 10847 | 6.2 | 1605 | 0.91 |  | - | - | - | - |  | 10847 | 1605 |
| Kidney | C64 | 3434 | 2.0 | 841 | 0.48 |  | 1992 | 1.1 | 474 | 0.26 |  | 5426 | 1315 |
| Bladder | C67 | 1024 | 0.57 | 253 | 0.14 |  | 709 | 0.39 | 146 | 0.08 |  | 1733 | 399 |
| Brain, central nervous system | C70-72 | 4320 | 2.5 | 2703 | 1.5 |  | 3359 | 1.9 | 2134 | 1.2 |  | 7679 | 4837 |
| Thyroid | C73 | 5303 | 3.0 | 159 | 0.09 |  | 25988 | 14.2 | 231 | 0.13 |  | 31291 | 390 |
| Hodgkin lymphoma | C81 | 2757 | 1.6 | 493 | 0.28 |  | 2094 | 1.2 | 378 | 0.21 |  | 4851 | 871 |
| Non-Hodgkin lymphoma | C82-86+C88 | 6209 | 3.5 | 2112 | 1.2 |  | 4240 | 2.3 | 1282 | 0.71 |  | 10449 | 3394 |
| Multiple myeloma | C90 | 845 | 0.49 | 403 | 0.23 |  | 568 | 0.31 | 279 | 0.15 |  | 1413 | 682 |
| Leukaemia | C91-95 | 5548 | 3.2 | 4079 | 2.4 |  | 4472 | 2.6 | 3241 | 1.8 |  | 10020 | 7320 |
