# Supplementary material for: Cancer Incidence and Mortality Estimates in Latin America and the Caribbean: A Systematic Analysis of the GLOBOCAN 2022
Source: Cancer Res Commun. 2025 Dec 29;5(12):2236–48. doi: 10.1158/2767-9764.CRC-25-0564 (PMC12745351; doi:10.1158/2767-9764.CRC-25-0564)
Supplement: Supplementary Table S3 — Table S3. Estimated incident early-onset cancer cases and deaths for 2022 and projected values for 2050, stratified by country and sex. [file crc-25-0564_supplementary_table_s3_suppst3.docx]

**Supplementary Table 3.** Estimated incident early-onset cancer cases and deaths for 2022 and projected values for 2050, stratified by country and sex.

| **Region** | **Male incidence** | |  | **Female incidence** | |  | **Male mortality** | |  | **Female mortality** | |
| --- | --- | --- | --- | --- | --- | --- | --- | --- | --- | --- | --- |
|  | 2022 estimated incidence | 2050 projected incidence |  | 2022 estimated incidence | 2050 projected incidence |  | 2022 estimated mortality | 2050 projected mortality |  | 2022 estimated mortality | 2050 projected mortality |
| Argentina | 7017 | 7800 |  | 15417 | 16385 |  | 2472 | 2806 |  | 3945 | 4255 |
| Bahamas | 58 | 62 |  | 109 | 123 |  | 11 | 12 |  | 37 | 44 |
| Barbados | 49 | 45 |  | 83 | 70 |  | 11 | 10 |  | 24 | 21 |
| Bolivia | 935 | 1448 |  | 3278 | 5083 |  | 515 | 809 |  | 1173 | 1897 |
| Brazil | 30630 | 29198 |  | 68986 | 62575 |  | 12334 | 11938 |  | 17702 | 16296 |
| Belize | 16 | 22 |  | 71 | 100 |  | 4 | 5 |  | 25 | 35 |
| Chile | 2371 | 2086 |  | 4262 | 3725 |  | 839 | 749 |  | 1126 | 1001 |
| Colombia | 6452 | 6501 |  | 14518 | 14158 |  | 2633 | 2688 |  | 3950 | 3913 |
| Costa Rica | 647 | 625 |  | 1415 | 1366 |  | 241 | 236 |  | 335 | 326 |
| Cuba | 1515 | 1193 |  | 2560 | 1973 |  | 564 | 445 |  | 692 | 538 |
| Dominican Republic | 1519 | 1820 |  | 2994 | 3509 |  | 609 | 739 |  | 1099 | 1316 |
| Ecuador | 1692 | 2043 |  | 4241 | 5169 |  | 767 | 934 |  | 1264 | 1568 |
| El Salvador | 599 | 697 |  | 1381 | 1342 |  | 287 | 339 |  | 474 | 469 |
| French Guyana | 30 | 52 |  | 63 | 104 |  | 6 | 10 |  | 11 | 18 |
| France, Guadeloupe | 59 | 71 |  | 103 | 101 |  | 20 | 24 |  | 23 | 22 |
| Guatemala | 1205 | 1858 |  | 3064 | 4707 |  | 664 | 1035 |  | 1190 | 1881 |
| Guyana | 43 | 55 |  | 163 | 184 |  | 27 | 34 |  | 58 | 66 |
| Haiti | 1177 | 1709 |  | 2041 | 2850 |  | 679 | 1023 |  | 1003 | 1452 |
| Honduras | 951 | 1403 |  | 2136 | 3178 |  | 674 | 994 |  | 971 | 1455 |
| Jamaica | 357 | 297 |  | 1004 | 761 |  | 151 | 130 |  | 390 | 312 |
| France, Martinique | 21 | 25 |  | 97 | 84 |  | 10 | 12 |  | 21 | 18 |
| Mexico | 15760 | 16463 |  | 32409 | 33646 |  | 5920 | 6304 |  | 8729 | 9236 |
| Nicaragua | 660 | 878 |  | 1314 | 1707 |  | 374 | 508 |  | 538 | 704 |
| Panama | 455 | 555 |  | 1148 | 1397 |  | 160 | 196 |  | 291 | 359 |

| Paraguay | 961 | 1131 |  | 2283 | 2715 |  | 333 | 406 |  | 614 | 761 |
| --- | --- | --- | --- | --- | --- | --- | --- | --- | --- | --- | --- |
| Peru | 4017 | 4649 |  | 9693 | 11348 |  | 1715 | 1990 |  | 2905 | 3454 |
| Puerto Rico | 510 | 474 |  | 926 | 791 |  | 125 | 121 |  | 140 | 122 |
| Saint Lucia | 19 | 18 |  | 55 | 46 |  | 2 | 2 |  | 11 | 10 |
| Suriname | 73 | 89 |  | 160 | 202 |  | 28 | 35 |  | 37 | 48 |
| Trinidad and Tobago | 224 | 200 |  | 457 | 399 |  | 60 | 54 |  | 127 | 112 |
| Uruguay | 677 | 642 |  | 1281 | 1185 |  | 199 | 193 |  | 278 | 262 |
| Venezuela | 3645 | 4899 |  | 7429 | 9731 |  | 1454 | 1974 |  | 2498 | 3307 |
