# Supplementary material for: Cancer Incidence and Mortality Estimates in Latin America and the Caribbean: A Systematic Analysis of the GLOBOCAN 2022
Source: Cancer Res Commun. 2025 Dec 29;5(12):2236–48. doi: 10.1158/2767-9764.CRC-25-0564 (PMC12745351; doi:10.1158/2767-9764.CRC-25-0564)
Supplement: Supplementary Table S4 — Table S4. Annual Percent Change (APC) in Cancer Mortality (1990–2022) for top 3 most incident early-onset cancers. [file crc-25-0564_supplementary_table_s4_suppst4.docx]

## **Supplementary Table 4.** Annual Percent Change (APC) in Cancer Mortality (1990–2022 ) for top 3 most incident early-onset cancers.

| Country | Male | | | Female | | |
| --- | --- | --- | --- | --- | --- | --- |
|  | Prostate | Colorectal | Lung | Breast | Colorectal | Lung |
| Chile | **-1.71 (-2.01 to -1.40)** | **1.44 (1.18 to 1.70)** | **-1.61 (-1.76 to -1.45)** | **-0.72 (-0.85 to -0.59)** | **0.89 (0.71 to 1.06)** | **1.04 (0.86 to 1.22)** |
| Colombia | -0.42 (-0.89 to 0.04) | **1.41 (1.24 to 1.57)** | **-1.49 (-1.84 to -1.15)** | **0.86 (0.69 to 1.03)** | **0.96 (0.75 to 1.17)** | **-0.64 (-0.97 to -0.31)** |
| Costa Rica | **-1.36 (-1.88 to -0.85)** | **1.27 (0.88 to 1.66)** | **-2.75 (-3.19 to -2.29)** | -0.13 (-0.42 to 0.16) | **0.63 (0.29 to 0.97)** | **-1.61 (-2.09 to -1.13)** |
| Cuba | **0.73 (0.55 to 0.91)** | **0.28 (0.04 to 0.52)** | **-0.43 (-0.62 to -0.25)** | -0.15 (-0.31 to 0.00) | 0.13 (0.00 to 0.26) | **1.21 (0.94 to 1.47)** |
| Argentina | **-1.52 (-1.80 to -1.24)** | **-0.31 (-0.44 to -0.18)** | **-2.50 (-2.68 to -2.33)** | **-0.92 (-1.01 to -0.83)** | **-0.19 (-0.29 to -0.09)** | **1.51 (1.32 to 1.70)** |
| Guatemala | **3.91 (2.93 to 4.91)** | **3.00 (2.22 to 3.79)** | -0.14 (-1.01 to 0.74) | **2.63 (2.05 to 3.21)** | **2.34 (1.80 to 2.88)** | 0.00 (-0.70 to 0.71) |
| Mexico | **0.26 (0.09 to 0.42)** | **2.48 (2.30 to 2.67)** | **-3.27 (-3.49 to -3.05)** | **0.55 (0.43 to 0.67)** | **1.34 (1.16 to 1.52)** | **-1.90 (-2.04 to -1.75)** |
| Paraguay | **0.66 (0.11 to 1.21)** | **3.33 (2.71 to 3.96)** | **0.74 (0.23 to 1.26)** | **1.76 (1.32 to 2.20)** | **2.97 (2.33 to 3.62)** | **1.31 (0.46 to 2.17)** |
| Brazil | **0.53 (0.03 to 1.03)** | **1.82 (1.69 to 1.95)** | **-0.95 (-1.13 to -0.77)** | **0.53 (0.46 to 0.59)** | **1.21 (1.08 to 1.34)** | **1.88 (1.71 to 2.05)** |
| USA | **-2.72 (-2.94 to -2.51)** | **-2.12 (-2.21 to -2.02)** | **-3.27 (-3.49 to -3.05)** | **-1.99 (-2.06 to -1.92)** | **-1.94 (-2.03 to -1.84)** | **-1.63 (-1.95 to -1.31)** |
| Uruguay | **-1.13 (-1.43 to -0.82)** | -0.10 (-0.31 to 0.12) | **-1.48 (-1.65 to -1.31)** | **-1.02 (-1.27 to -0.77)** | **-0.82 (-1.01 to -0.62)** | **3.61 (3.16 to 4.08)** |

Values represent Annual Percent Change (APC) and 95% Confidence Intervals (CI) in age-standardized mortality rates (1990–2022). Bold indicates statistical significance (p < 0.05).
