# Supplementary material for: Cancer Incidence and Mortality Estimates in Latin America and the Caribbean: A Systematic Analysis of the GLOBOCAN 2022
Source: Cancer Res Commun. 2025 Dec 29;5(12):2236–48. doi: 10.1158/2767-9764.CRC-25-0564 (PMC12745351; doi:10.1158/2767-9764.CRC-25-0564)
Supplement: Supplementary Table S5 — Table S5. Linear Regression Between Human Development Index (HDI) and Cancer Burden Indicators (1990–2022) [file crc-25-0564_supplementary_table_s5_suppst5.docx]

## **Supplementary Table 5.** Linear Regression Between Human Development Index (HDI) and Cancer Burden Indicators (1990–2022)

| **Indicator** | **Intercept (β₀)** | **Slope (β₁, HDI)** | **Std. Error** | **R²** | **95% CI for R** | **p-value** |
| --- | --- | --- | --- | --- | --- | --- |
| ASIR (Age-Standardized Incidence Rate) | 37.91 | 66.15 | 40.66 | 0.09 | 0.00–0.59 | 0.115 |
| ASMR (Age-Standardized Mortality Rate) | −82.34 | 344.84 | 74.34 | 0.43 | 0.15–0.72 | **7.51×10⁻⁵** |
| MIR (Mortality-to-Incidence Ratio) | 0.996 | −0.656 | 0.126 | 0.492 | 0.46–0.85 | **1.6×10⁻⁵** |

β₁ represents the estimated change in the outcome per unit increase in HDI. Bolded values indicate statistical significance (p < 0.05). MIR = ASMR / ASIR.
