# Supplementary material for: Cancer Incidence and Mortality Estimates in Latin America and the Caribbean: A Systematic Analysis of the GLOBOCAN 2022
Source: Cancer Res Commun. 2025 Dec 29;5(12):2236–48. doi: 10.1158/2767-9764.CRC-25-0564 (PMC12745351; doi:10.1158/2767-9764.CRC-25-0564)
Supplement: Supplementary Table S6 — Table S6. Estimated incident cancer cases and deaths for 2022 and projected values for 2050, stratified by cancer type and sex. [file crc-25-0564_supplementary_table_s6_suppst6.docx]

**Supplementary Table 6.** Estimated incident cancer cases and deaths for 2022 and projected values for 2050, stratified by cancer type and sex.

| **Cancer Type** | **Male incidence** | |  | **Female incidence** | |  | **Male mortality** | |  | **Female mortality** | |
| --- | --- | --- | --- | --- | --- | --- | --- | --- | --- | --- | --- |
|  | 2022 estimated incidence | 2050 projected incidence |  | 2022 estimated incidence | 2050 projected incidence |  | 2022 estimated mortality | 2050 projected mortality |  | 2022 estimated mortality | 2050 projected mortality |
| Lip, oral cavity | 13098 | 22541 |  | 6153 | 12087 |  | 5697 | 10289 |  | 2625 | 5530 |
| Salivary glands | 2549 | 4610 |  | 1870 | 3343 |  | 881 | 1755 |  | 548 | 1154 |
| Oropharynx | 7766 | 12972 |  | 1726 | 3135 |  | 4368 | 7728 |  | 925 | 1849 |
| Nasopharynx | 1577 | 2587 |  | 636 | 1059 |  | 899 | 1553 |  | 358 | 634 |
| Hypopharynx | 2139 | 3823 |  | 361 | 644 |  | 949 | 1752 |  | 143 | 282 |
| Oesophagus | 15454 | 29121 |  | 4861 | 9732 |  | 14430 | 27637 |  | 4417 | 8994 |
| Stomach | 45056 | 92323 |  | 29201 | 59459 |  | 35027 | 72750 |  | 22776 | 47172 |
| Liver and intrahepatic bile ducts | 22889 | 46733 |  | 19775 | 41759 |  | 21059 | 43609 |  | 18200 | 39000 |
| Gallbladder | 2988 | 6110 |  | 7437 | 14319 |  | 2105 | 4367 |  | 4938 | 9715 |
| Pancreas | 20180 | 40281 |  | 20764 | 42481 |  | 18827 | 38026 |  | 19413 | 40222 |
| Larynx | 14270 | 26300 |  | 2980 | 5439 |  | 9006 | 17099 |  | 1424 | 2818 |
| Trachea, bronchus and lung | 61777 | 123176 |  | 43175 | 83066 |  | 53311 | 108224 |  | 37239 | 73064 |
| Melanoma of skin | 10679 | 20226 |  | 9599 | 16663 |  | 3280 | 6657 |  | 2560 | 5181 |
| Non-melanoma skin cancer | 43182 | 93465 |  | 29643 | 66960 |  | 4589 | 10603 |  | 3484 | 8664 |
| Mesothelioma | 767 | 1572 |  | 441 | 820 |  | 710 | 1477 |  | 399 | 760 |
| Kaposi sarcoma | 2387 | 3364 |  | 426 | 660 |  | 452 | 639 |  | 98 | 171 |
| Penis | 5185 | 10028 |  | - | - |  | 1674 | 3173 |  | - | - |
| Prostate | 225316 | 464415 |  | - | - |  | 60792 | 145428 |  | - | - |
| Testis | 13644 | 14913 |  | - | - |  | 2103 | 2497 |  | - | - |
| Kidney | 23032 | 40891 |  | 12851 | 22290 |  | 10066 | 19729 |  | 5594 | 10897 |
| Bladder | 25521 | 54088 |  | 10188 | 20558 |  | 9804 | 22378 |  | 4100 | 9182 |
| Brain, central nervous system | 14296 | 22220 |  | 12651 | 19996 |  | 11901 | 20027 |  | 10595 | 18064 |
| Thyroid | 11946 | 17210 |  | 51533 | 68027 |  | 1518 | 3115 |  | 3081 | 6642 |
| Hodgkin lymphoma | 5774 | 8112 |  | 4259 | 6030 |  | 1619 | 2755 |  | 1212 | 2121 |
| Non-Hodgkin lymphoma | 24038 | 40904 |  | 19005 | 33190 |  | 10730 | 20068 |  | 8481 | 16610 |
| Multiple myeloma | 8395 | 16038 |  | 6758 | 12688 |  | 6215 | 12532 |  | 5154 | 10219 |
| Leukaemia | 22504 | 35665 |  | 18421 | 28914 |  | 15670 | 26948 |  | 12942 | 22007 |
| Colorectum | 72752 | 139824 |  | 72017 | 136849 |  | 36956 | 74890 |  | 36490 | 73882 |
| Breast | - | - |  | 219684 | 341863 |  | - | - |  | 59701 | 45161 |
| Vulva | - | - |  | 3647 | 7359 |  | - | - |  | 1359 | 1679 |
| Vagina | - | - |  | 1615 | 2980 |  | - | - |  | 572 | 616 |
| Cervix uteri | - | - |  | 63056 | 97236 |  | - | - |  | 33443 | 23890 |
| Corpus uteri | - | - |  | 34612 | 58462 |  | - | - |  | 9250 | 8433 |
| Ovary | - | - |  | 24031 | 38619 |  | - | - |  | 15881 | 12252 |
