# Supplementary material for: Cancer Incidence and Mortality Estimates in Latin America and the Caribbean: A Systematic Analysis of the GLOBOCAN 2022
Source: Cancer Res Commun. 2025 Dec 29;5(12):2236–48. doi: 10.1158/2767-9764.CRC-25-0564 (PMC12745351; doi:10.1158/2767-9764.CRC-25-0564)
Supplement: Supplementary Figure S1 — Figure S1. ASIR and ASMR in 2022 for all countries in patients with early-onset cancer. [file crc-25-0564_supplementary_figure_s1_suppsf1.docx]

**Supplementary Figure 1.** ASIR and ASMR in 2022 for all countries in patients with early-onset cancer. (A) LAC map of ASIR for both sexes. (B) LAC map displaying ASMR for both sexes. (C) Bar plot of ASIR stratified by sex and country. (D) Bar plot of ASMR stratified by sex and country.

**
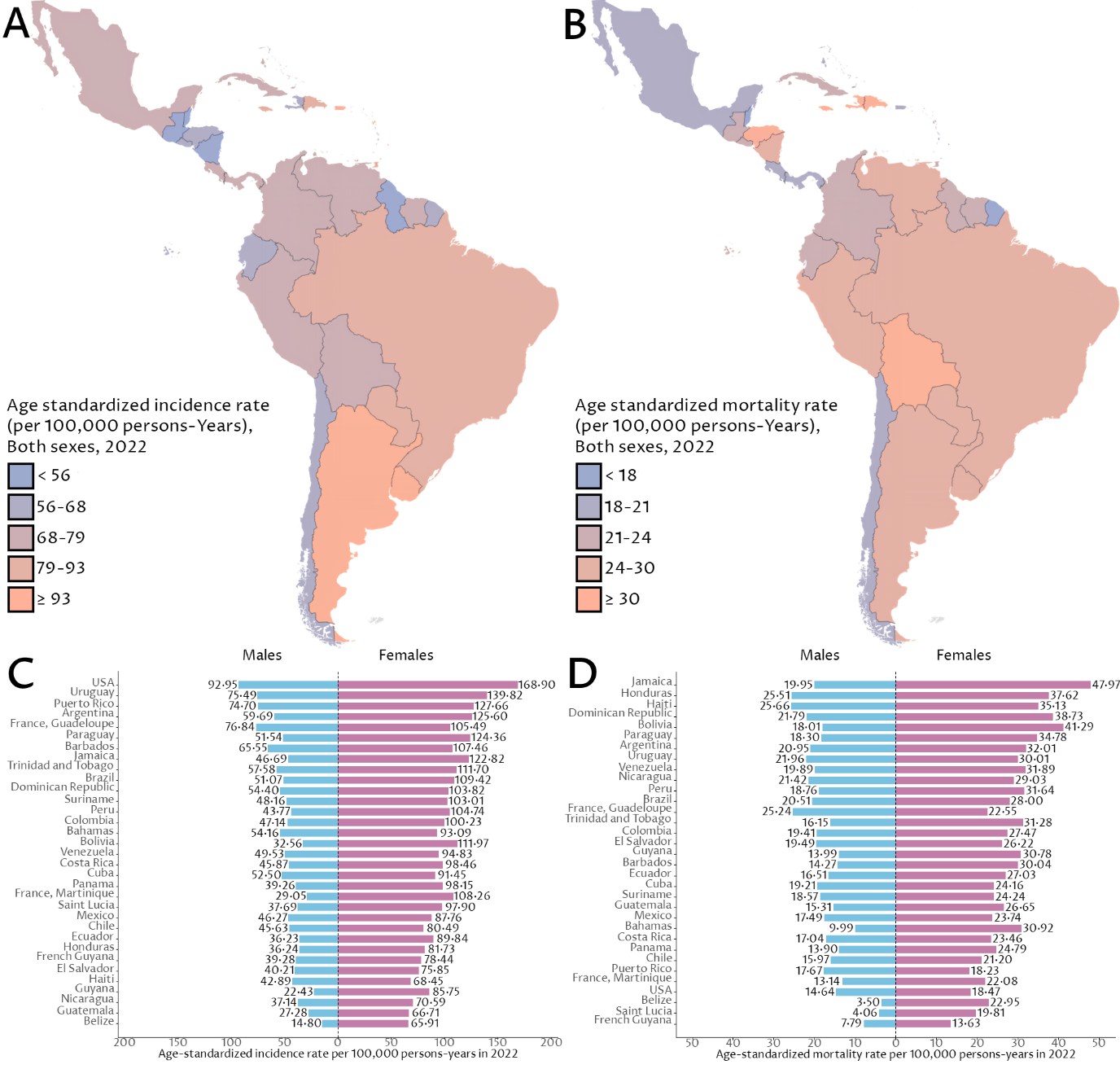
**
