# Supplementary material for: Cancer Incidence and Mortality Estimates in Latin America and the Caribbean: A Systematic Analysis of the GLOBOCAN 2022
Source: Cancer Res Commun. 2025 Dec 29;5(12):2236–48. doi: 10.1158/2767-9764.CRC-25-0564 (PMC12745351; doi:10.1158/2767-9764.CRC-25-0564)
Supplement: Supplementary Figure S2 — Figure S2. Cancer types by ASIR and ASMR in 2022 in patients with early-onset cancer. [file crc-25-0564_supplementary_figure_s2_suppsf2.docx]

**
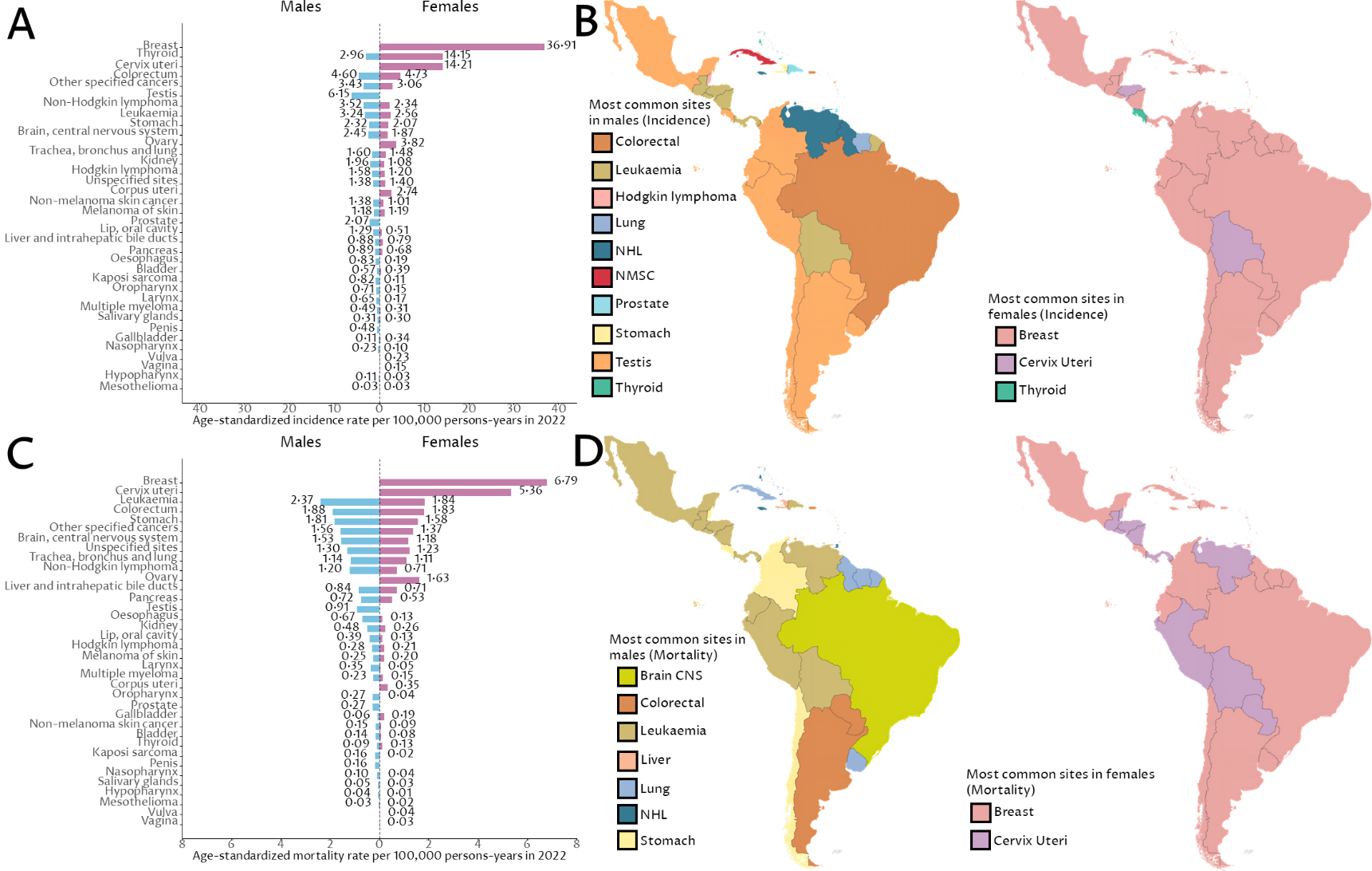
Supplementary Figure 2.** Cancer types by ASIR and ASMR in 2022 in patients with early-onset cancer. (A) Bar plot of ASIR stratified by sex and cancer type. (B) Sex-stratified maps of LAC showing the most common incidence sites by country. (C) Bar plot of ASMR stratified by sex and cancer type. (D) Sex-stratified maps of LAC displaying the most frequent mortality sites by country.
