# Supplementary material for: Cancer Incidence and Mortality Estimates in Latin America and the Caribbean: A Systematic Analysis of the GLOBOCAN 2022
Source: Cancer Res Commun. 2025 Dec 29;5(12):2236–48. doi: 10.1158/2767-9764.CRC-25-0564 (PMC12745351; doi:10.1158/2767-9764.CRC-25-0564)
Supplement: Supplementary Figure S3 — Figure S3. Projected cases and deaths numbers (per 1,000 persons) under varying global rate-change scenarios in patients with early-onset cancer, 2022–2050. [file crc-25-0564_supplementary_figure_s3_suppsf3.docx]

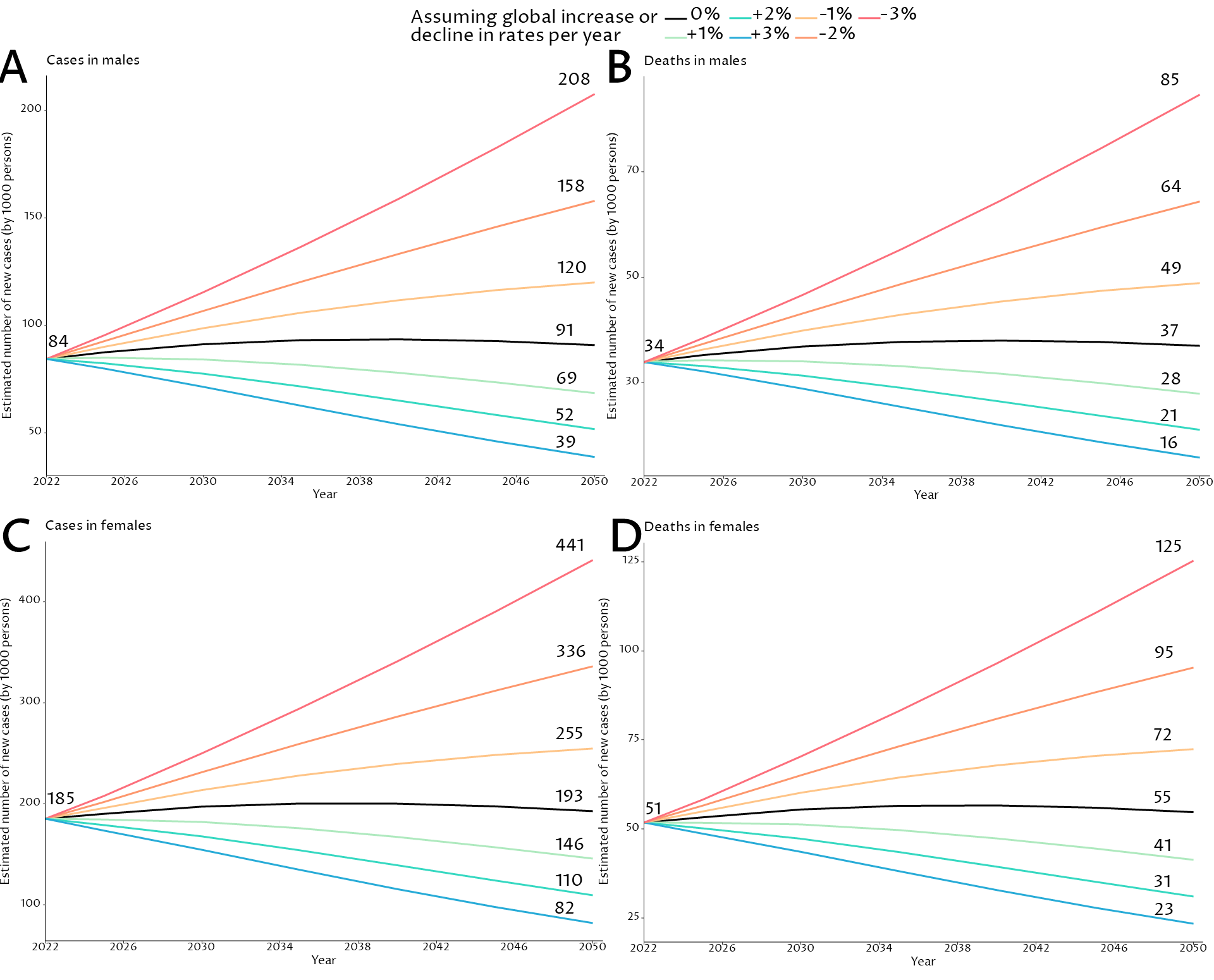
**Supplementary Figure 3.** Projected numbers of cases and deaths (per 1,000 persons) under various global rate-change scenarios for patients with early-onset cancer from 2022 to 2050. (A) Incidence projections for males. (B) Mortality projections for males. (C) Incidence projections for females. (D) Mortality projections for females.
