# Supplementary material for: Cancer Incidence and Mortality Estimates in Latin America and the Caribbean: A Systematic Analysis of the GLOBOCAN 2022
Source: Cancer Res Commun. 2025 Dec 29;5(12):2236–48. doi: 10.1158/2767-9764.CRC-25-0564 (PMC12745351; doi:10.1158/2767-9764.CRC-25-0564)

**Supplementary Figure 4.** ASMR in LAC countries by age group and sex. All sites excluding non-melanoma skin cancer, 1990–2022. (A) All ages - males. (B) All ages - females. (C) Early-onset - males. (D) Early-onset - females.


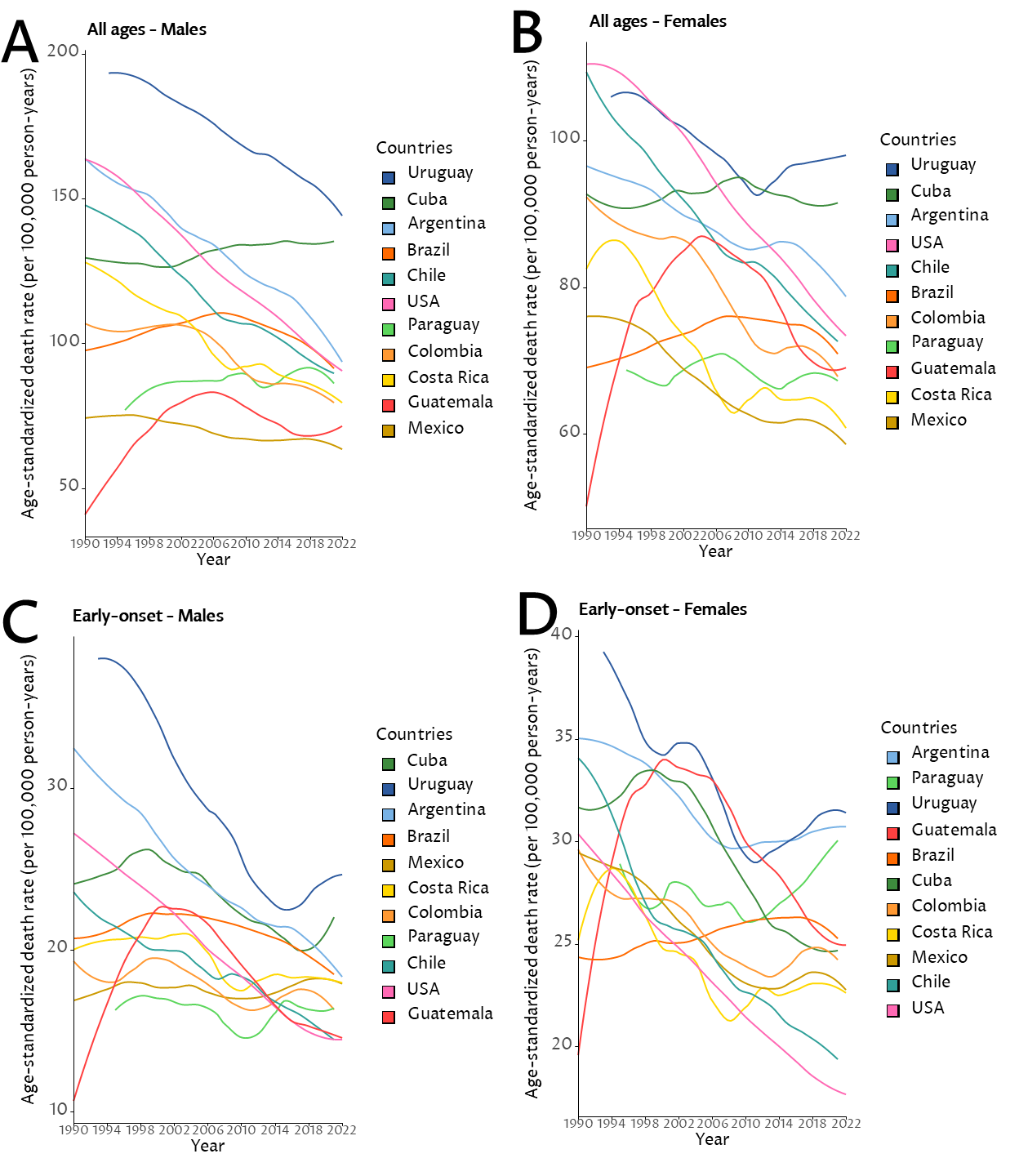

Supplement: Supplementary Figure S4 — Figure S4. ASMR in LAC countries by age group and sex. All sites excluding non-melanoma skin cancer, 1990–2022. [file crc-25-0564_supplementary_figure_s4_suppsf4.docx]
