# Supplementary material for: Cancer Incidence and Mortality Estimates in Latin America and the Caribbean: A Systematic Analysis of the GLOBOCAN 2022
Source: Cancer Res Commun. 2025 Dec 29;5(12):2236–48. doi: 10.1158/2767-9764.CRC-25-0564 (PMC12745351; doi:10.1158/2767-9764.CRC-25-0564)

**Supplementary Figure 5.** ASMR in LAC countries by cancer type in patients with early-onset cancer, 1990–2022. (A) Prostate cancer - males. (B) Breast cancer - females. (C) Colorectal cancer - males. (D) Colorectal cancer - females. (E) Lung cancer - males. (F) Lung cancer - females.


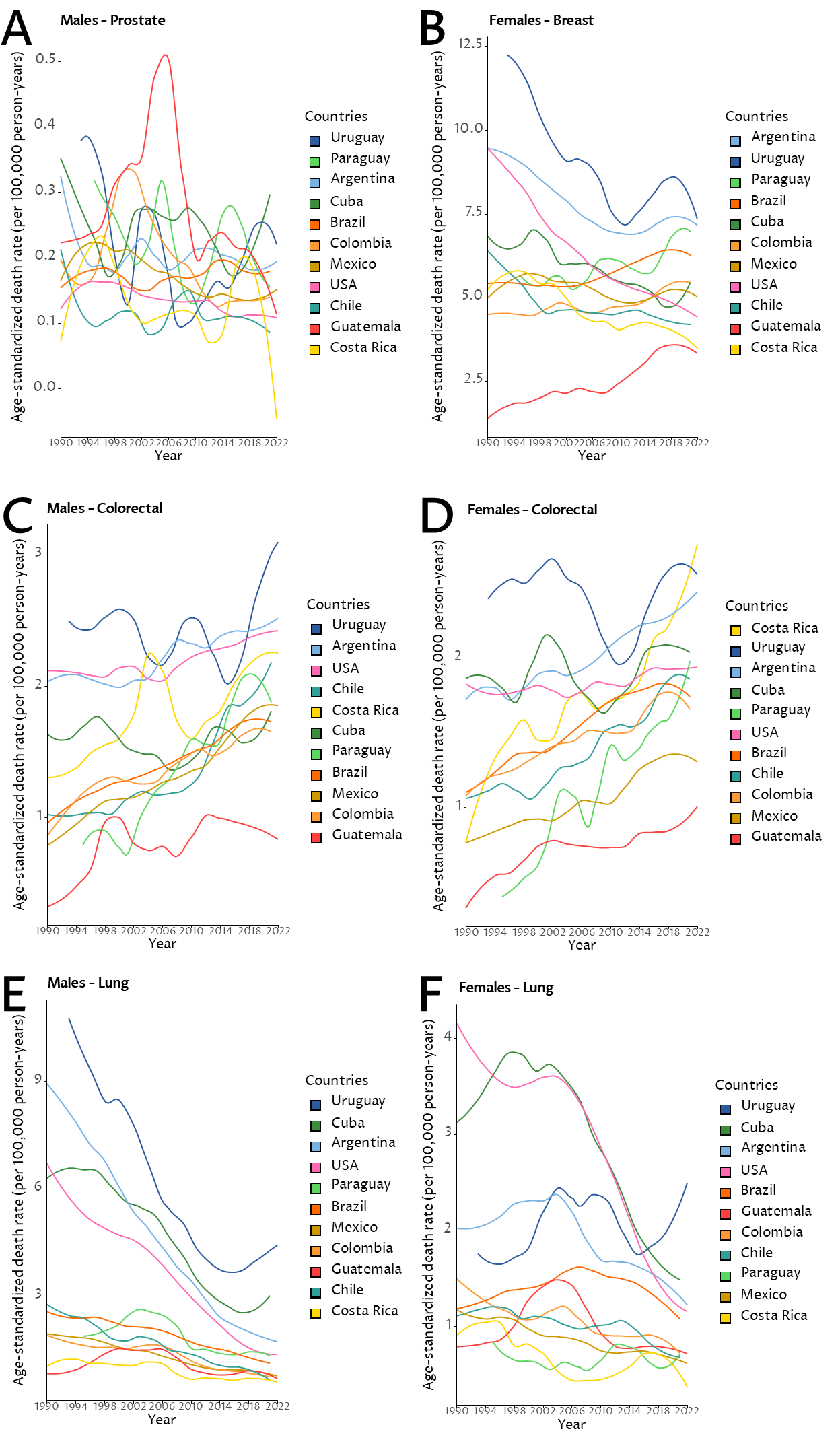

Supplement: Supplementary Figure S5 — Figure S5. ASMR in LAC countries by cancer type in patients with early-onset cancer, 1990–2022. [file crc-25-0564_supplementary_figure_s5_suppsf5.docx]
