# Supplementary material for: Cancer Incidence and Mortality Estimates in Latin America and the Caribbean: A Systematic Analysis of the GLOBOCAN 2022
Source: Cancer Res Commun. 2025 Dec 29;5(12):2236–48. doi: 10.1158/2767-9764.CRC-25-0564 (PMC12745351; doi:10.1158/2767-9764.CRC-25-0564)
Supplement: Supplementary Figure S6 — Figure S6. Bar plots of ASIR and ASMR in 2022 for all countries and cancer types in patients with 15-39 years. [file crc-25-0564_supplementary_figure_s6_suppsf6.docx]

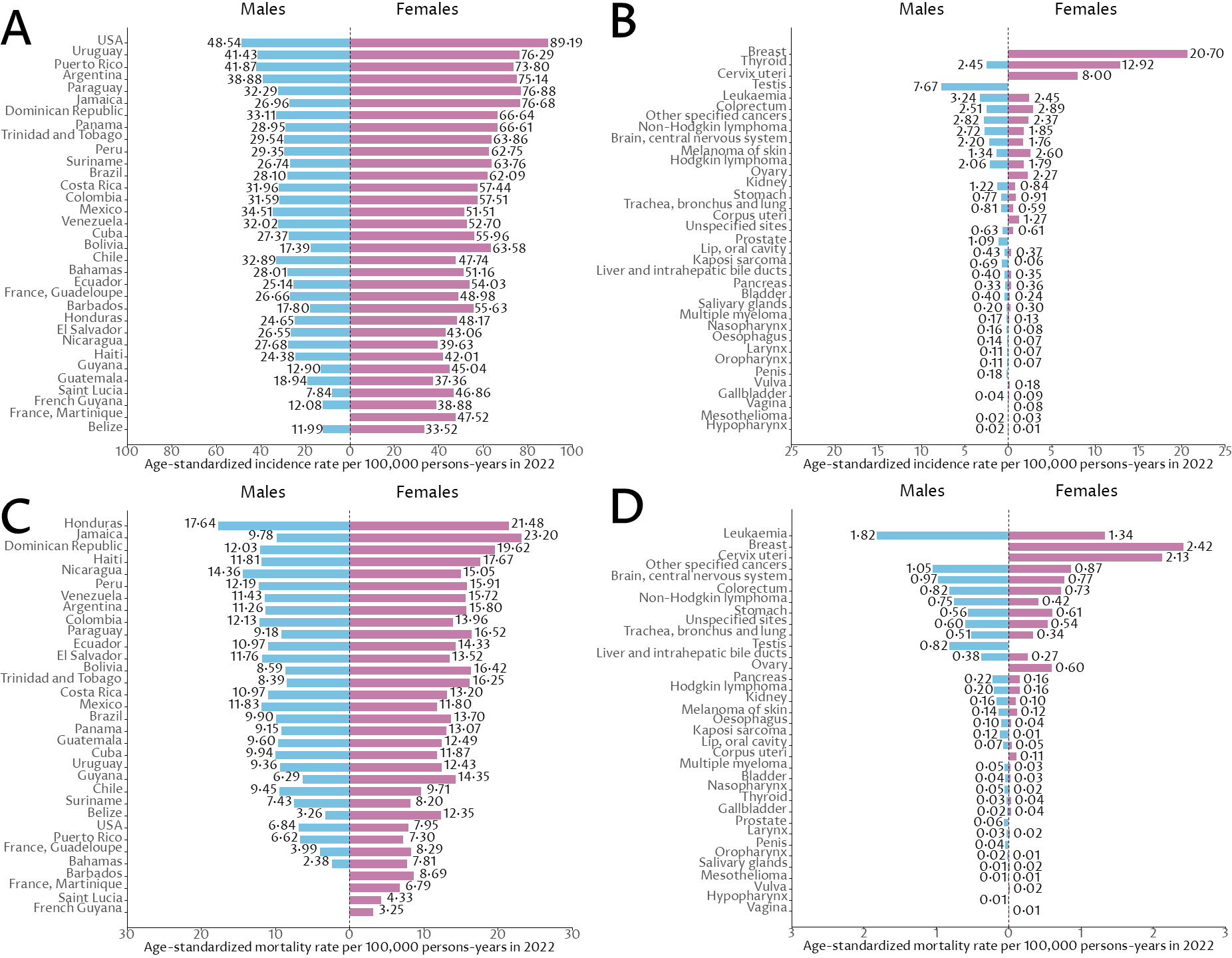
**Supplementary Figure 6.** Bar plots of ASIR and ASMR in 2022 for all countries and cancer types in patients with 15-39 years. (A) ASIR stratified by sex and country. (B) ASIR stratified by sex and cancer type. (C) ASMR stratified by sex and country. (D) ASMR stratified by sex and cancer type.
