# Supplementary material for: Cancer Incidence and Mortality Estimates in Latin America and the Caribbean: A Systematic Analysis of the GLOBOCAN 2022
Source: Cancer Res Commun. 2025 Dec 29;5(12):2236–48. doi: 10.1158/2767-9764.CRC-25-0564 (PMC12745351; doi:10.1158/2767-9764.CRC-25-0564)

**Supplementary Figure 1.** ASIR and ASMR in 2022 for all countries in patients with early-onset cancer. (A) LAC map of ASIR for both sexes. (B) LAC map displaying ASMR for both sexes. (C) Bar plot of ASIR stratified by sex and country. (D) Bar plot of ASMR stratified by sex and country.

**Supplementary Figure 7.** Projected cases and deaths numbers (per 1,000 persons) under varying global rate-change scenarios, 2022–2050. (A) Cases projections in females. (B) Cases projections in males. (C) Death projections in females. (D) Death projections in males.


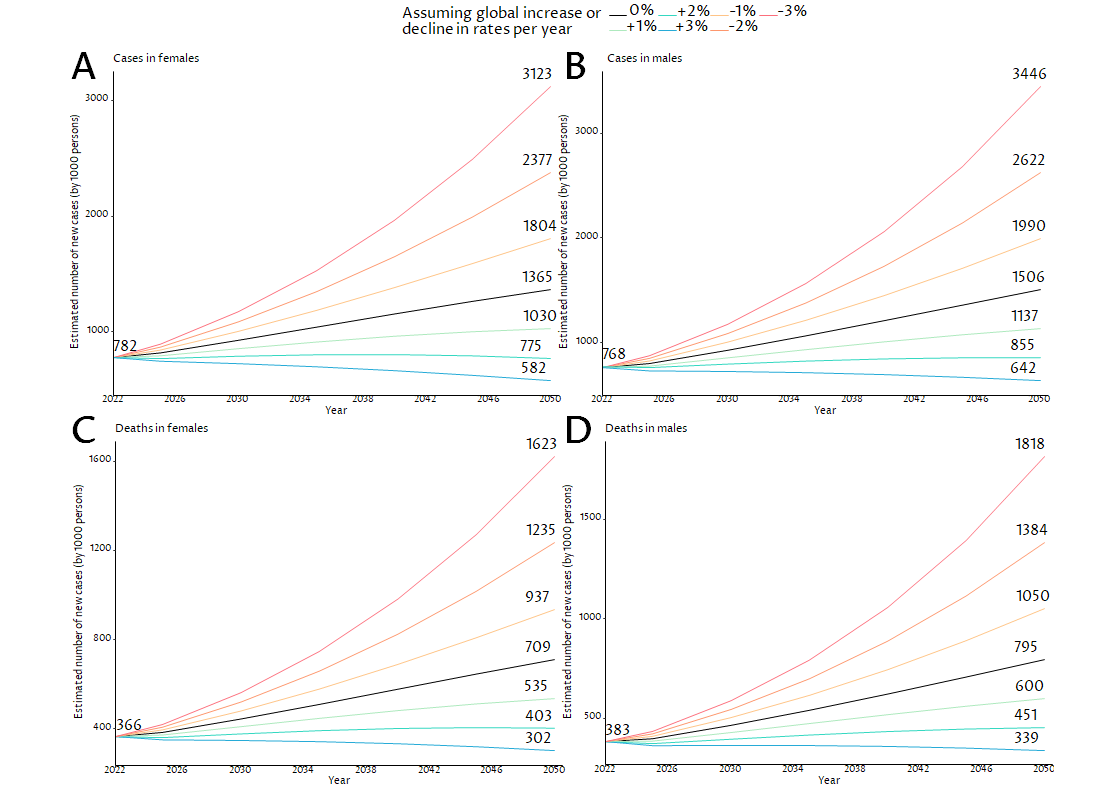

Supplement: Supplementary Figure S7 — Figure S7. Projected cases and deaths numbers (per 1,000 persons) under varying global rate-change scenarios, 2022–2050. [file crc-25-0564_supplementary_figure_s7_suppsf7.docx]
